# Synthesis, cytotoxicity, pharmacokinetic profile, binding with DNA and BSA of new imidazo[1,2-*a*]pyrazine-benzo[*d*]imidazol-5-yl hybrids

**DOI:** 10.1038/s41598-020-63605-4

**Published:** 2020-04-16

**Authors:** Iqubal Singh, Vijay Luxami, Kamaldeep Paul

**Affiliations:** School of Chemistry and Biochemistry, Thapar Institute of Engineering and Technology, Patiala, 147001 India

**Keywords:** DNA, Drug discovery and development

## Abstract

Novel derivatives possessing imidazo[1,2-*a*]pyrazine and 1*H*-benzo[*d*]imidazole scaffolds were synthesized using Suzuki-Miyaura cross-coupling reactions. *In vitro* anticancer activities against NCI-60 cancer cell panels were tested at 10 *µ*M concentration. The best results were obtained from substitution of two 1-cyclohexyl-1*H*-benzo[*d*]imidazole groups present at C-6 and C-8 positions of imidazo[1,2-*a*]pyrazine (**31**). Compound **31** was found to be cytotoxic against 51 cell lines and cytostatic against 8 cell lines with broad range of growth inhibitions (−98.48 to 98.86%). GI_50_ value of compound **31** was found in the range of 0.80–2.87 *µ*M for 59 human cancer cell lines at five-dose concentration levels. DNA binding study of potent compound **31** was suggested that this compound was intercalated into DNA base pairs with binding constant of 1.25 × 10^4^ M^−1^. Compound **31** showed effective binding with bovine serum albumin (BSA) and presented binding constant value of 3.79 ×10^4^ M^-1^. Pharmacokinetic studies revealed that all compounds are following Lipinski’s rule of five and expected to be orally active.

## Introduction

Cancer is one of the major reasons for death globally. Millions of people suffer or die from cancer each year, and there is no particular good medication available at present^1^. Cancer has significant economic impact, which is increasing gradually. In the field of cancer treatment, chemists have great challenges to discover new and efficient compounds with strong and broad-spectrum anticancer activity. Heterocyclic ring systems play significant role in the discovery of novel bioactive substances due to their minimal side effects and effective at small doses. Heterocyclic moieties are widely present in various natural bioactive compounds^2^. Undesirable side effects, toxicity, drug resistance, and low bioavailability are some of the major known problems for currently available anticancer agents^3^. Therefore, there is an urgent need for the discovery of more efficient and selective anticancer agent.

The biological potential of these heterocycles towards cancer cells has been stated with diverse mechanism of action. Most of the anticancer candidates bind with DNA double-strand and interfere with replication and transcription, thus, interrupt DNA function and alter the cell division. These candidates show interaction between adjacent base pairs or bind with grooves of DNA. These candidates share similar structural features, for example, the presence of planarity in structure, which can help the molecule to bind with DNA through insertion between base-pairs. The basic chain connected with these heterocyclic moieties plays a significant role in the selectivity and affinity^4^. Due to their high affinity, the discovery of novel DNA intercalator for cancer therapy is a crucial goal in the medicinal chemistry. These intercalators comprise anthracyclines^5,6^ (e.g. doxorubicin and mitoxantrone), ellipticine^7^ and acridine derivatives (e.g. amsacrine)^8^, used in the cancer therapy of acute leukemia, breast, and ovarian cancers.

On account of reported pharmacophoric features of DNA intercalators, we used a structure-based drug designing technique that includes the combination of two or more heterocyclic moieties with related biological activities. This may agree on the development of new imidazo[1,2-*a*]pyrazine-benzimidazole derivatives, having basic pharmacophoric features of anticancer agents and DNA binders.

Considering the importance of these moieties, seventeen imidazo[1,2-*a*]pyrazine-benzimdazole derivatives (**9-23**, **30** and **31**) were synthesized. Structures of all newly synthesized derivatives were uploaded to National Cancer Institute (NCI), Bethesda, Maryland, USA. Amongst these derivatives, nine compounds (**8-10**, **12-14**, **22**, and **30-31**) were selected for their *in vitro* evaluation against cancer cell lines at 10 *μ*M concentration level. Compound **31**, with two 1-cyclohexyl-1*H*-benzo[*d*]imidazole groups at C-6 and C-8 positions of imidazo[1,2-*a*]pyrazine, has been shown broad-spectrum towards anticancer activity than other groups in all cancer cell lines. Therefore, compound **31** was further used to investigate the interaction with calf thymus (CT)-DNA. Bovine serum albumin (BSA) studies were also investigated to find out the transportation properties. Pharmacokinetic properties of all synthesized compounds were evaluated to ensure significant oral bioavailability.

## Results and Discussion

### Chemistry

Suzuki-Miyaura cross-coupling approach has been employed for the synthesis of imidazo[1,2-*a*]pyrazine–benzimidazole hybrids (**9-23**, **30** and **31**) and outlined in Figs. 2 and 3. Nitration of 1,4-dibromobenzene **1** afforded intermediate **2** in 93% yield followed by nucleophilic substitution with cyclohexylamine in the presence of K_2_CO_3_ in DMF at 100 °C for 18 h to obtain 4-bromo-*N*-cyclohexyl-2-nitroaniline **3** in 75% yield. Formation of intermediate **3** was supported by NMR spectral analysis, the characteristic multiplet at δ 3.52–3.43 ppm for one proton and signals ranging from 2.14 to 1.25 for ten protons corresponding to cyclohexyl ring. Boronation of intermediate **3** with bis(pinacolato)diboron in 1,4-dioxane in the presence of Pd(PPh_3_)_2_Cl_2_ and KOAc afforded product **4** in 82% yield. Compound **4** was further characterized by NMR with characteristic signals of four methyl groups of boronate having singlet of 12 protons at δ 1.32 ppm.

**Figure 1:**
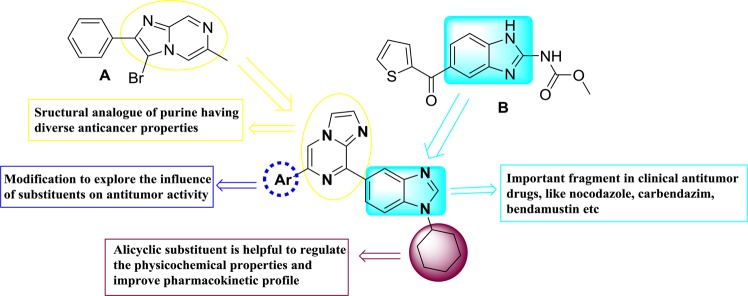
Rationale for the designing of benzimidazoles-imidazo[1,2-*a*]pyrazine hybrids^32,33^. Over the past years, benzimidazole derivatives have been widely studied for their antimalarial^34^, anticancer^35^, antiprotozoal^36^, anti-inflammatory, and analgesic^37^ activities. On the other hand, imidazo[1,2-*a*]pyrazine is also reported as cyclic nucleotide phosphodiesterase inhibitor^38^, anticancer^39^, anti-inflammatory^40^, antioxidant^41^, antimicrobial^42^, antiviral^43^, and antimalarial agents^44^. Thus, the designing of benzimidazole-imidazo[1,2-*a*]pyrazine hybrid ring system has been taken that provides new compounds related to various biological activities of benzimidazole and imidazopyrazine, in the hope that new anti-tumor agents might be discovered. Alicyclic substitution at NH of benzimidazole moiety can enhance the pharmacokinetic properties of the scaffold. By taking these perspectives into consideration, the lead molecule was designed. To explore the effect of substitution on cytotoxicity, a library of compounds was synthesized by modification of different substitution on phenyl ring and benzimidazole moiety (Fig. 1).

**Figure 2:**
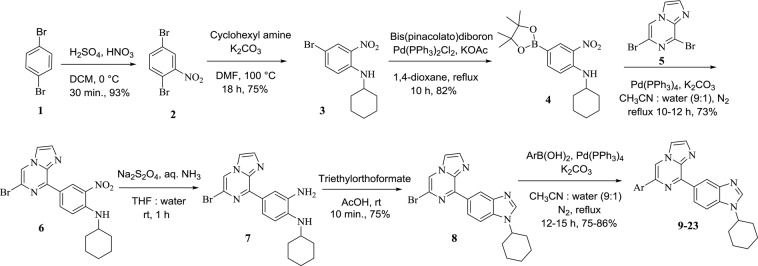
Synthesis of 6-substituted-8-(1-cyclohexyl-1*H*-benzo[*d*]imidazol-5-yl)imidazo[1,2-*a*]pyrazine.

**Figure 3:**
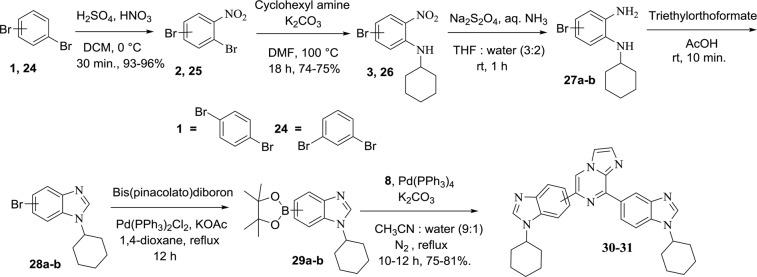
Synthesis of bisbenzimidazole derivatives **30** and **31**.

Under Suzuki-coupling conditions *viz*., Pd(PPh_3_)_4_, K_2_CO_3_ in CH_3_CN and water, intermediate **4** was reacted with dibromo imidazo[1,2-*a*]pyrazine **5** to yield 4-(6-bromoimidazo[1,2-*a*]pyrazin-8-yl)-*N*-cyclohexyl-2-nitroaniline **6** in 73% yield. Reduction of **6** was carried out using sodium dithionite to afford compound **7** followed by cyclization in acetic acid and triethyl orthoformate produced intermediate **8** in 75% yield. Compound **8** was confirmed by ^1^H NMR with shifting of multiplet to downfield at δ 4.26–4.18 ppm for one proton corresponding to CH of cyclohexyl and absence of NH signal at δ 8.40 ppm. Compound **8** was further used for Suzuki reactions with substituted phenyl, thienyl and naphthyl boronates to give **9-23** in 75–86% yields (Fig. 2).

The synthesis of bisbenzimidazole (benzimidazole at C-6 and C-8 positions of imidazo[1,2-*a*]pyrazine) has been shown in Fig. 3. First, nitration of 1,3-dibromobenzene with nitric acid afforded derivative **25** in 96% yield followed by treatment with cyclohexylamine in the presence of K_2_CO_3_ in DMF at 100 °C yielded aminated precursor **26** in quantitative yield. Derivatives **3** and **26** were then reduced with sodium dithionite in THF and water to afford intermediate **27a-b**. Derivatives **27a-b** were cyclized using triethyl orthoformate in the presence of acetic acid to get benzimidazole **28a-b**. Boronation was carried out with bis(pinacolato)diboron using Pd(PPh_3_)_2_Cl_2_ and KOAc in dioxane afforded intermediate **29a-b**. Suzuki-Miyaura cross-coupling of intermediates **29a** and **29b** with **8** were accomplished using Pd(PPh_3_)_4_ and K_2_CO_3_ to obtain **30** and **31** in 75% and 81% yields, respectively. Compounds **30** and **31** were distinguished by difference in the signal of CH corresponding to cyclohexyl group in ^1^H NMR. All the final compounds were confirmed by NMR and mass spectrometry (Figs S1–S54).

### Cytotoxicity

#### Cytotoxicity at one dose concentration (10 *µ*M)

**Figure 4:**
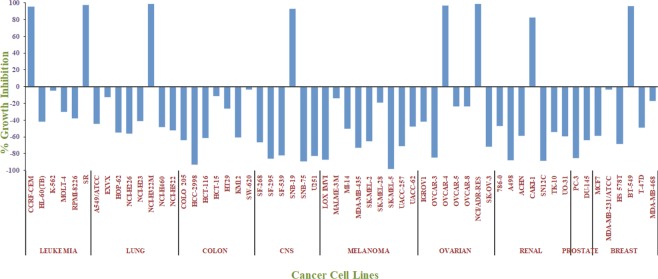
The inhibitory effects of compound **31** on human cancer cell lines at single concentration of 10 *µ*M.

Amongst all newly synthesized derivatives, nine compounds were selected by NCI for their *in-vitro* cytotoxicity at single dose concentration (10 *µ*M) towards sixty subcancer cell lines of nine main panels. Analyzed all compounds exhibited diverse activity for different cancer cells (Table S1). The bisbenzimidazole derivatives **30** and **31** with benzimidazole rings at C6 and C8 positions of imidazo[1,2-*a*]pyrazine showed promising cytotoxicity against human cancer cell lines. Compound **31** exhibited a broad spectrum of activity and showed sensitivity against all 59 tested cancer cell lines. Derivative **31** has been observed as most potent derivative in each group of cancer. Derivative **31** was found to be cytotoxic effect against 51 cell lines and cytostatic effect towards 8 cell lines with a broad range of growth inhibition (−98.48 to 98.86%). SK-MEL-5 (melanoma) and HCC-2998 (colon cancer) cell lines have been found to be the most sensitive cell lines for compound **31** (Fig. 4). Compound **30** showed maximum inhibitory effect against RPMI-8226 (leukemia) amongst all cancer cell lines with growth inhibition of 52.18%. About 50% growth inhibition for HL-60 (TB) and MOLT-4 (leukemia) cancer cell lines was observed for compound **12** having naphthalene substitution at the C6 position of imidazo[1,2-*a*]pyrazine. Similarly, 3-thiophene substitution (compound **14**) displayed more than 50% growth inhibition against T-47D (breast cancer) cancer cell lines.

Interestingly, among all evaluated compounds, compound **31** was found to be most active in the preliminary test at single dose concentration and thus preceded to five dose concentration assays.

#### Cytotoxicity at five dose concentrations (0.01–100 *µ*M)

*In vitro* screening of compound **31** against full panel of 60 cancer cell lines at five different concentrations has been performed and results are shown in Table 1. Compound **31** exhibited broad spectrum of growth inhibition for nine panels of cell lines with GI_50_ values in the range of 0.80–2.87 *µ*M and full panel mean graph mid-point (MG-MID) to be 2.12 *µ*M. Compound **31** was observed to be sensitive towards most of the given cell lines and showed excellent activity with RPMI-8226 of leukemia having GI_50_ value of 806 nM.

**Table 1:** Cytotoxicity of compound **31** at five dose concentration level (0.01–100 *µ*M).

Cell Panel	Cell Line	GI_50_ (*µ*M)	TGI (*µ*M)	LC_50_ (*µ*M)
Leukemia	CCRF-CEM	1.69	6.08	>1.00
	HL-60(TB)	1.84	4.57	>1.00
	K-562	2.57	>1.00	>1.00
	MOLT-4	1.64	5.22	>1.00
	RPMI-8226	0.80	3.99	>1.00
	SR	2.87	>1.00	>1.00
Non-Small Cell Lung Cancer	A549/ATCC	1.94	3.54	6.46
	EXVX	1.97	NT	>1.00
	HOP-62	2.00	3.72	6.90
	HOP-92	1.54	3.22	6.73
	NCI-H226	1.69	3.79	NT
	NCI-H23	2.01	4.09	NT
	NCI-H322M	1.91	4.14	NT
	NCI-H460	1.98	4.02	8.16
	NCI-522	1.74	3.40	NT
Colon Cancer	COLO 205	1.93	3.79	7.44
	HCC-2998	1.81	3.46	659
	HCT-116	1.70	NT	NT
	HCT-15	2.07	NT	>1.00
	HT29	2.44	5.20	>1.00
	KM12	2.01	3.83	NT
	SW-620	2.08	3.97	NT
CNS Cancer	SF-268	1.59	3.15	6.23
	SF-295	1.71	3.15	5.80
	SF-539	1.63	3.15	6.10
	SNB-19	2.11	4.95	>1.00
	SNB-75	1.39	3.07	6.77
	U251	1.91	3.65	6.98
Melanoma	LOX IMVI	1.68	3.22	NT
	MALME-3M	2.11	3.75	6.68
	M14	1.77	3.65	NT
	MDA-MB-435	1.77	3.55	7.11
	SK-MEL-2	1.74	3.35	6.45
	SK-MEL-28	1.81	3.41	6.43
	SK-MEL-5	1.49	2.83	5.39
	UACC-257	2.10	3.62	6.24
	UACC-62	1.70	3.36	NT
Ovarian Cancer	IGROV1	2.04	NT	NT
	OVCAR-3	1.83	3.36	6.20
	OVCAR-4	2.17	6.30	>1.00
	OVCAR-5	2.43	NT	>1.00
	OVCAR-8	2.55	9.49	>1.00
	NCI/ADR-RES	2.34	7.33	>1.00
	SK-OV-3	2.27	4.10	7.42
Renal Cancer	786-0	1.73	3.47	NT
	A498	1.77	3.22	5.85
	ACHN	1.68	3.07	5.61
	CAKI-1	1.46	2.95	5.97
	RXF 393	1.51	2.99	5.94
	SN12C	2.05	4.53	>1.00
	TK-10	2.02	3.65	NT
	UO-31	1.49	2.85	5.46
Prostate Cancer	PC-3	1.70	3.93	NT
	DU-145	1.77	3.28	NT
Breast Cancer	MCF7	1.80	4.58	>1.00
	MDA-MB-231/ATCC	1.29	3.08	7.33
	HS 578 T	2.39	6.74	>1.00
	BT-549	1.94	3.64	6.82
	T-47D	1.90	4.69	>1.00
	MDA-MB-468	2.32	NT	>1.00
Full panel mean-graph midpoint (MIG-MID)		2.12	4.73	6.63

NT = not tested; MG-MID = average sensitivity of derivative against all cancer cell (*µ*M); GI_50_ = concentration of the derivative required for 50% of maximal growth inhibition; TGI = concentration of the derivative required for total growth inhibition; LC_50_ = concentration of the derivative required to kill 50% of population.

#### Cytotoxicity at normal cell line

**Figure 5:**
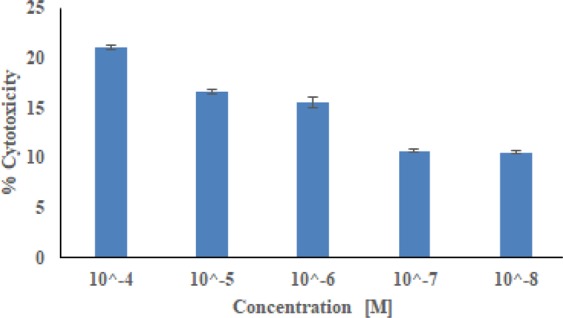
Cytotoxicity of derivative **31** against human normal cell line Hek293 (All experiments were performed in triplicates at five different concentrations 10^−4^ to 10^−8^ M. The Microsoft Office Professional Plus 13 (excel) was used to plot the graph, and to calculate standard deviation).

To check the safety profile, compounds **31** was evaluated against Human Embryonic Kidney cells (HEK293) through MTT assay. The results indicated that compound has not shown any significant toxicity against HEK293 cells at 10^−4^, 10^−5^, 10^−6^, 10^−7^ and 10^−8^ M concentrations, suggesting great potential for their *in-vivo* use as antitumor agents (Fig. 5). It was found that on treating the cells with compound **31**, the % survivals of HEK293 cells were well above 79%, 83%, 84%, 89% and 90% at 10^−4^, 10^−5^, 10^−6^, 10^−7^ and 10^−8^ M concentrations, respectively. This clearly indicated that these compounds were well within the toxicity limits and thereby, exhibiting good safety profile and have a high prospective for *in vivo* use as antitumor agents.

### DNA binding studies

DNA is a key drug target and numerous derivatives show their antitumor activity by binding to DNA and interfering with DNA replication and preventing the growth of cancer cells, which is the foundation of designing novel and potent anticancer agents. The efficiency of DNA targeting drug depends upon its binding mode and affinity^9^. Thus, DNA binding study of small molecule is essential in the development of novel therapeutic substance^10^. Therefore, the interaction of compound **31** with DNA was analyzed by a number of techniques, such as UV-Vis absorption, fluorescence, and circular dichroism spectroscopy.

#### Absorption spectral studies

**Figure 6:**
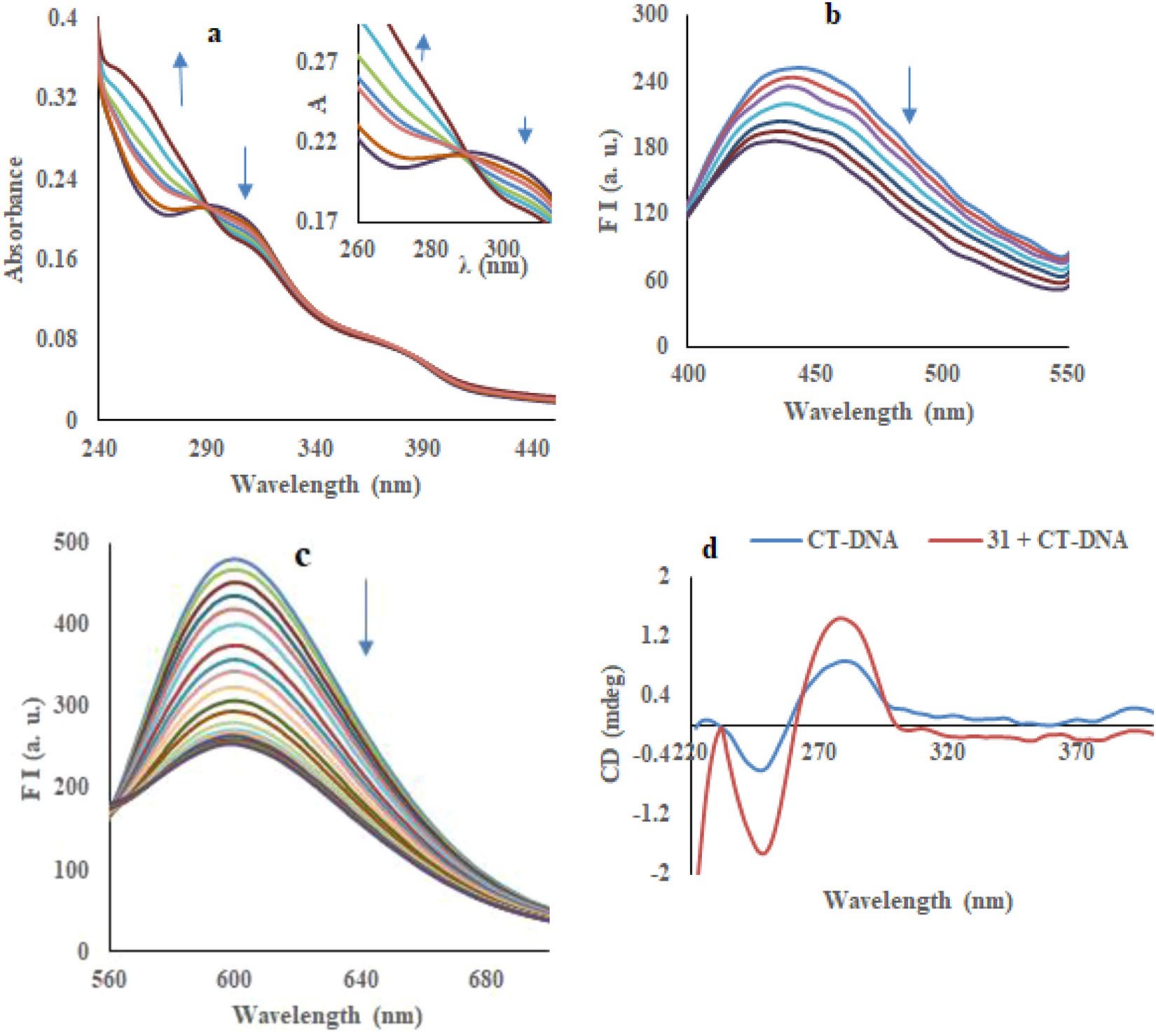
(**a**) Electronic spectrum of compound **31** with incremental addition of CT-DNA; (**b**) Fluorescence quenching of compound **31** with increasing concentration of CT-DNA; (**c**) Emission spectra of EtBr-CT-DNA complex with increasing concentration of compound **31**; (**d**) CD spectra of free CT-DNA (blue) and CT-DNA-derivative **31** complex (red).

The electronic absorption spectrum of compound **31** (20 *µ*M) consists of band in the range of 270–400 nm in phosphate buffer (*p*H 7.4). Compound **31** showed the high energy absorption band in the spectrum at 290 nm. Upon increasing concentrations of CT-DNA (0–15 *µ*M), the band at 290 nm showed hypochromism (Fig. 6a). Titration was extended until the saturation point. These results suggested that the compound used in this study showed binding to DNA in an intercalative mode. To find the binding efficiency of derivative **31**, binding constant (K_b_) with CT-DNA has been obtained by applying the Benesi-Hildebrand equation (Eq. 1)^11^ and calculated to be 1.25 × 10^4^ M^−1^ (Table 2) **(**Figure S55). The observed value of binding constant (K_b_) revealed that compound **31** was effectively bound with DNA.

**Table 2:** Binding parameters of compound **31** upon CT-DNA and BSA interactions.

Biomolecules	K_SV_ (×10^4^) (M^−1^)	K_q_ (×10^12^) (M^−1^s^−1^)	^a^R	K_bin_ (×10^4^ M^−1^)	n	^b^R
CT-DNA	0.93	0.93	0.9942	9.58	1.21	0.9630
BSA	10.40	10.40	0.9825	4.70	0.92	0.9797

^a^R (K_SV_ and K_q_) and ^b^R (K_bin_ and n) are the correlation coefficients.

#### Fluorescence spectral studies

To investigate the effect of adding DNA on compound **31**, luminescence study has been used to get the information about binding constant and binding mode. The fluorescence spectrum of compound **31**, exhibiting an emission band at 445 nm (λ_ex_ = 295 nm), was monitored at a fixed concentration of 5 *µ*M in phosphate buffer having *p*H 7.4 at 298 K. On addition of CT-DNA (0–50 *µ*M) into solution of compound **31**, gradual quenching of fluorescence intensity by 75% was detected (Fig. 6b). The result proposes that the quenching of fluorescence intensity of compound **31** might be due to the interaction between CT-DNA and compound. Stern-Volmer equation (equation-2) was applied to calculate the value of K_sv_ (Stern-Volmer constant) and K_q_ (apparent bimolecular quenching constant)^12^. The value of K_sv_ (Stern-Volmer constant) has been obtained from the slope and was found to be 93 × 10^2^ M^−1^ (Figure S56a). Linear Stern-Volmer plot showed a single quenching process, either static or dynamic. The value of K_q_ has been calculated using τ_o_ (lifetime of the fluorophore)^13^ = 10^−8^ s and was found to be 93 **×** 10^10^ M^−1^ s^−1^ (Table 2). The value of K_q_ is greater than maximum dynamic quenching constant (~1 × 10^10^ M^−1^ s^−1^)^14^, represents that interaction of DNA with compound **31** is probably involved the static quenching mechanism.

The binding constant (K_bin_) and the number of binding sites (n) for static quenching interaction of DNA with compound **31** were determined by Scatchard equation (Eq. 3).^15^ The calculated value of K_bin_ = 9.58 × 10^4^ M^−1^ suggested that derivative **31** has strong binding affinity towards DNA. The number of binding sites (n) was obtained to be 1.21, indicating 1:1 stoichiometry between compound **31** and DNA (Figure S56b).

#### Competitive binding between compound 31 and ethidium bromide for CT-DNA

Competitive binding experiment with compound **31** was conducted to get more evidence for the binding of the compound to DNA. Ethidium bromide (EtBr) shows no emission band in the buffer^16^. EtBr displayed enhanced emission band at 606 nm upon intercalating with CT-DNA, when excited at 520 nm. The EtBr-CT-DNA (3 *µ*M: 30 *µ*M) complex showed significant quenching after addition of compound **31** (0-80 *µ*M) due to the displacement of EtBr from DNA, suggesting strong binding of compound **31** to DNA (Fig. 6c). The detected linearity in the plot of F_o_/F *versus* concentration of compound **31** is in good agreement with linear Stern-Volmer equation (Eq. 2)^12^. The Stern-Volmer plot (Figure S57) has been used to determine the quenching constant and was calculated to be 1.16 × 10^4^ M^−1^. The calculated apparent binding constant value [K_app_ = 7.50 × 10^5^ M^−1^] for compound **31** revealed effective EtBr-displacement ability of compound and strong binding to DNA (Eq. 4).

#### Thermal denaturation studies

A thermal denaturation experiment was performed to get further insight into binding mode of compound **31** with DNA. The observed ∆*T*_*m*_ value of 12 °C in the presence of compound **31** demonstrated that binding of compound stabilized the DNA double helix. An increase in the melting temperature of CT-DNA in the presence of compound also supported the intercalative mode of binding for compound **31**.

#### Circular dichroism (CD) spectroscopy

To further understand the changes of polynucleotide properties prompted by compound **31**, and with the aim to demonstrate the intercalation between base pairs of DNA, CD technique was used. Circular dichroism spectrum of CT-DNA displayed two peaks; the first negative peak at 249 nm and the second positive band at 280 nm as the results of right-handed helicity and base stacking, respectively^17^. The addition of compound **31** to DNA increased the intensities of 249 and 280 nm signals without any shift in their positions. Compound **31** also showed a weak negative induced CD (ICD) signal at 320–400 nm as a result of intercalation^18^. These observed results are in agreement with an intercalative binding of compound **31** with DNA (Fig. 6d).

### Bovine serum albumin (BSA) binding interaction

Serum albumins are the major plasma proteins and impart crucial role in nutrients and exogenous drug transport to the cells and tissues, and their metabolism^19^. Serum albumins are important blood proteins that have their ability to transport multitude of ligands. The binding ability of drug-albumin in the bloodstream has a significant impact on distribution, free concentration, metabolism and toxicity of drug^20^. Optimal interactions of any bioactive compound with serum albumin may increase drug efficiency. Bovine serum albumin (BSA) is the most extensively studied serum albumin owing to its structurally similar to human serum albumin.

#### Absorption spectroscopic studies

**Figure 7:**
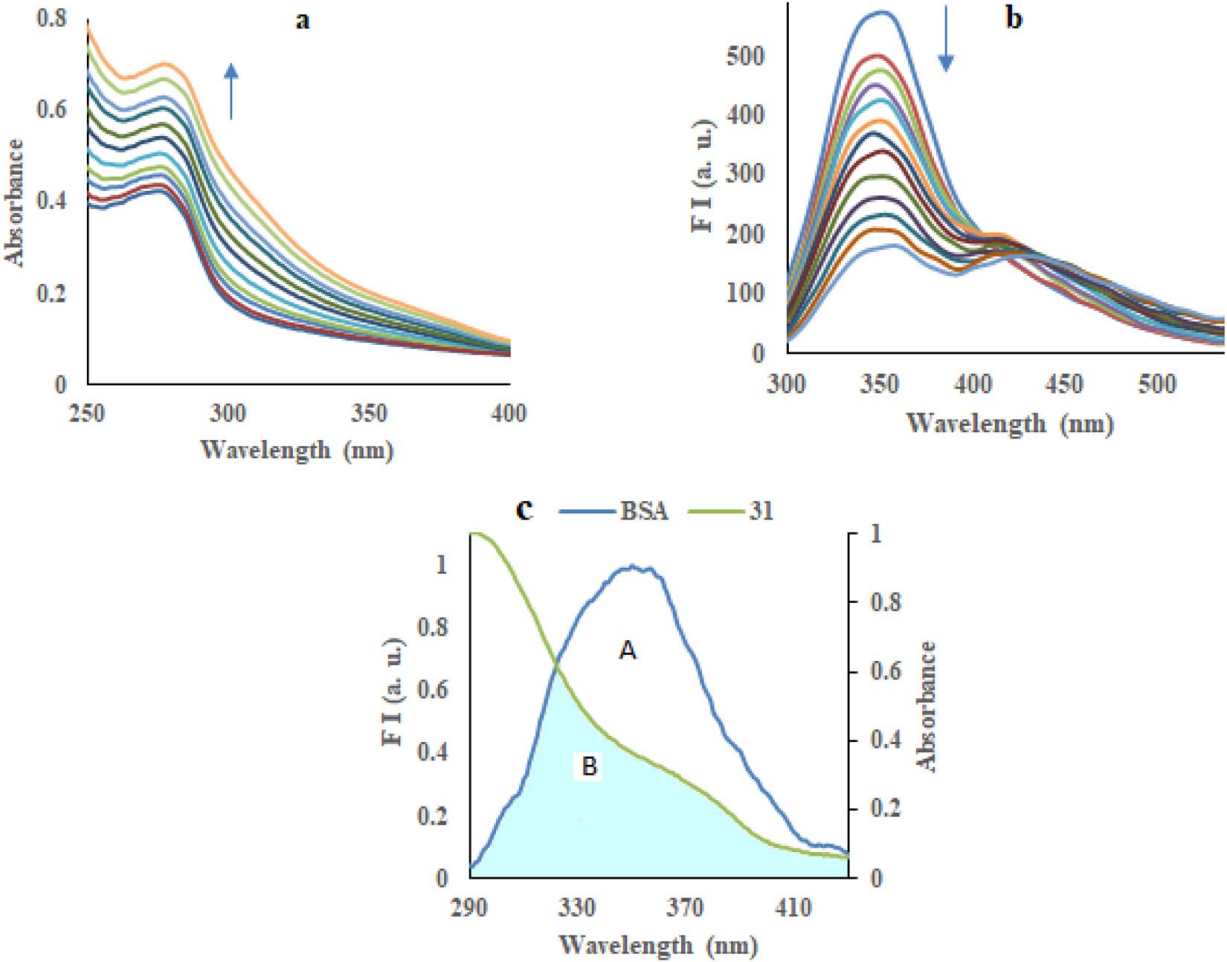
(**a**) Electronic spectrum and (**b**) fluorescence quenching of BSA with increasing concentration of compound **31**; (**c**) Overlap of absorption spectrum of derivative **31** with the emission spectrum of BSA.

To find the interaction between compound **31** and BSA, UV-Vis spectra were recorded. The absorption spectrum of a fixed amount of BSA (10 *µ*M) in phosphate buffer of *p*H 7.4 has been recorded with increasing concentration of compound **31** (0–8 *µ*M). The absorption spectrum of BSA showed a band at 280 nm as the results of aromatic amino acids present in structure of BSA. An increase in intensity of the absorption band at 280 nm has been achieved with incremental addition of derivative **31** without affecting position of band (Fig. 7a). These changes were obtained due to the variations in the conformation of BSA along with changes in the microenvironment polarity of aromatic residues, indicating interaction between compound **31** and BSA protein. To check the binding affinity of compound **31** with BSA, binding constant (K_b_) was determined by the Benesi-Hildebrand equation (Eq. 1)^11^ and calculated to be 3.79 ×10^4^ M^-1^ (Figure S58).

#### Fluorescence quenching studies with BSA

Bovine serum albumin binding affinity with compound **31** was examined by emission quenching of tryptophan. BSA shows an emission band near 350 nm, due to the presence of trp-212 and trp-134 residues in its structure, on excitation at 295 nm. A solution of fixed amount of BSA (10 *µ*M) when titrated with incremental addition of compound **31** (0–16 *µ*M), displayed quenching of emission band at 350 nm up to 70% of the initial fluorescence intensity of BSA (Fig. 7b). A hypsochromic shift (5 nm) was appeared as a result of formation of complex between compound **31** and BSA. The Stern-Volmer quenching constant (K_sv_) and apparent bimolecular quenching constant (K_q_) of compound **31** have been calculated with Stern-Volmer equation (Eq. 2)^12^ and were found to be 1.04 ×10^5^ M^−1^ and 1.04 × 10^13^ M^−1^s^−1^_,_ respectively (Table 2) (Figure S59a). The calculated value of K_sv_ for compound **31** is demonstrating promising binding affinity of compound for serum albumin. The linearity of the Stern-Volmer plot denotes single quenching phenomenon, either static (complex formation by quencher and fluorophore) or dynamic (collision process)^14^. A higher value of K_q_ indicating the existence of static quenching phenomena and the formation of the complex between compound **31** and BSA.

The binding constant (K_bin_) and the number of binding sites (n) for interaction of compound **31** with BSA have been obtained from the Scatchard equation (Eq. 3)^15^ and were calculated to be 4.70 × 10^4^ M^−1^ and 0.92, respectively (Table 2) (Figure S59b). The value of binding constant (K_bin_) suggested that BSA has a good affinity to compound **31**, as the known K_bin_ value of non-covalent interaction between BSA and drug is generally in the range of 10^4^–10^6^ M^−1^. The binding constant for interaction of compound **31** and BSA recommended that compound can easily be transported by the protein.

In order to estimate the distribution of compound **31**, complex of compound **31** (10 *µ*M) and BSA (10 *µ*M) was titrated with increasing concentration of ibuprofen (0-15 *µ*M). On excitation at 280 nm, the emission band of complex at 350 nm was effectively quenched by the addition of ibuprofen (Figure S60a). Results showed that ibuprofen efficiently displayed compound **31** from the complex. The dissociation constant for compound **31** was calculated using the Stern-Volmer equation (Eq. 2) and found to be 1.07 × 10^5^ M^−1^ (Figure S60b). The calculated dissociation constant was found equal to quenching constant for compound **31** that concludes the potential dissociation of compound **31** from BSA.

#### Förster resonance energy transfer (FRET) studies

To determine the spatial distances between the donor (BSA) and the acceptor (compound **31**), Förster resonance energy transfer (FRET) phenomenon was used^21^. According to the fundamental Förster mechanism, the energy transfer occurs when emission spectrum of the donor (BSA) overlap with the absorption spectrum of the acceptor (compound **31**). The fluorescence energy transfer method can be used to determine the Förster distance (r) between BSA and compound **31**. Figure 7c showed the overlap pattern of UV-visible absorption spectrum of compound **31** with emission spectrum of BSA. According to Eqs. 5 to 7, the parameters were calculated as the overlap integral value (*J*) between the emission spectrum of donor (BSA) and the absorption spectrum of acceptor (compound **31**) = 5.35 × 10^−15^ cm^3^ L mol^−1^, critical distance (R_o_) = 2.29 nm, energy transfer efficiency (*E*) = 64% (0.64), and distance between BSA and the compound **31** (r) = 2.08 nm. The distance between BSA and compound **31** (r) is less than 10 nm which is full agreement with the rule 0.5 R_o_ < r < 1.5 R_o_. Thus, the transfer of energy from BSA to derivative **31** must occur with high probability^22^.

### Physicochemical properties evaluation

Significant pharmacokinetic properties of any drug candidate can overcome the problem of failure of clinical trials during development. The rule-of–five (RO5) deals with orally active candidates and describe various ranges of four physicochemical factors (log *P* ≤ 5, Mol. Wt ≤ 500, no. of H-bond acceptors ≤ 10 and no. of H-bond donors ≤ 5) for suitable aqueous solubility and intestinal permeability^23^. We have studied all the synthesized compounds (**9–23**, **30** and **31**) for these pharmacokinetic parameters. These compounds were found to have experimental log *P* values less than five and (Eq. 8) have molecular weight less than 500 except compounds **30** and **31**. All compounds have found number of hydrogen bond acceptors less than 10 and the number of hydrogen bond donors less than 5, according to the rule-of-five. It was also observed that these compounds showed % absorbance (ABS) in the range of 86.54–92.43% (Table 3) (Eq. 9), indicating good bioavailability. These observations projected that these compounds are expected to be orally active because they are following the parameters of Lipinski’s rule of five.

**Table 3:** Pharmacokinetic properties of compounds 9-23, 30 and 31.

Comp.	Ar	Exp. Log P	TPSA(Å)	MW	nON	nOHNH	nviolat.	nrot	%ABS
9		1.59	48.02	393	5	0	0	3	92.43
10		1.44	57.26	423	6	0	0	4	89.24
11		0.23	65.10	421	6	0	0	4	86.54
12		1.48	48.02	443	5	0	0	3	92.43
13		1.26	48.02	411	5	0	0	3	92.43
14		1.63	48.02	399	5	0	0	3	92.43
15		1.54	48.02	407	5	0	0	3	92.43
16		1.74	48.02	421	5	0	0	4	92.43
17		1.41	65.10	435	6	0	0	4	86.54
18		1.34	48.02	427	5	0	0	3	92.43
19		2.05	48.02	472	5	0	0	3	92.43
20		1.88	57.26	423	6	0	0	4	89.24
21		1.77	48.02	411	5	0	0	3	92.43
22		1.37	48.02	399	5	0	0	3	92.43
23		1.67	48.02	461	5	0	0	4	92.43
30		2.16	65.85	515	7	0	1	4	86.28
31		1.77	65.85	515	7	0	1	4	86.28

TPSA = Total polar surface area, nON = no. of hydrogen acceptors, nOHON = no. of hydrogen donors, nrot = number of rotational bonds, % ABS = percentage of absorbance.

### Molecular docking studies

**Figure 8:**
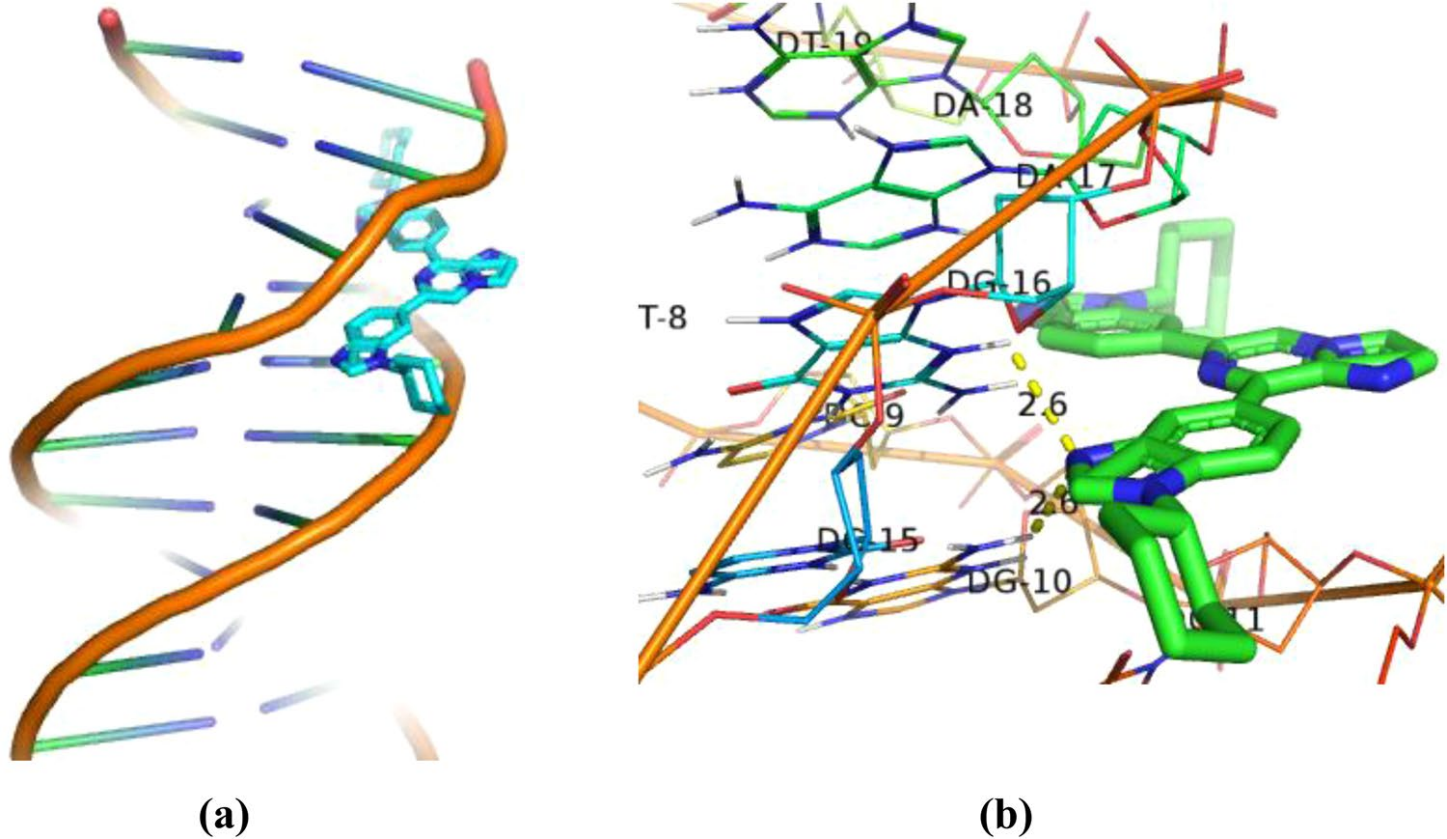
The complex of 31-DNA (PDB ID: 1BNA); (**a**) Surface representation; (**b**) Stereo view showing the possibility of hydrogen bonds with base pair of DNA) obtained by means of molecular docking.

With a view to confirm and rationalize the experimental results, interaction of compound **31** with DNA (PDB ID: 1BNA)^24^ has been investigated by molecular docking studies using Autodock 4.0^25^. Derivative **31** gave −11.1 Kcal/mol minimum binding energy on docking with DNA (Table S2). The docking studies of derivative **31** with DNA exhibited hydrogen bonding (*d* = 2.60 Å) interaction between nitrogen of benzimidazole ring and hydrogen (H-21) atom linked with nitrogen (N-2) of guanine (DG-10) of chain A. Similarly, compound **31** also showed hydrogen bonding (*d* = 2.60 Å) interaction of nitrogen of benzimidazole ring with H-3 of nitrogen (N-3) of guanine base (DG-16) present in chain B (Fig. 8).

## Conclusion

A new series of 17 compounds (**9-23**, **30** and **31**) possessing imidazo[1,2-*a*]pyrazine and 1-cyclohexyl-1*H*-benzo[*d*]imidazole scaffold was synthesized using the Suzuki-Miyaura cross-coupling approach. Nine compounds (**8-10**, **12-14**, **22**, **30** and **31**) were tested at single dose of 10 *µ*M at NCI over 60 human cell line panel, amongst which compound **31** was subsequently tested at five-dose concentration levels. Compound **31** demonstrated potent and broad-spectrum anticancer activity over all the tested nine cancer types. Compound **31** was found to be cytotoxic against 51 cell lines and cytostatic against 8 cell lines with a broad range of growth inhibition (−98.48 to 98.86%). Compound **31** exhibited a broad spectrum of growth inhibition for nine panels of cell lines with GI_50_ values in the range of 0.80–2.87 *µ*M. Compound **31** showed strong interaction with CT-DNA by intercalation binding mode with a binding constant value of 1.25 × 10^4^ M^−1^. Compound **31** also exhibited strong affinity towards bovine serum albumin (BSA) with binding constant of 3.79 ×10^4^ M^-1^. From Forster’s non-radiative energy transfer equations, it has been found that the distance of compound from BSA is 2.08 nm which predicts the possibility of energy transfer. All the compounds showed good pharmacokinetic properties and are expected to be orally active. Docking studies showed exceptional binding energy of DNA (PDB: 1BNA) with derivative **31** that has been found to be −11.1 Kcal/mol.

## Experimental protocol

### Cytotoxicity

Cytotoxicity was performed at National Cancer Institute (NCI), Bethesda, USA as per their protocols^26^.

### MTT assay

HEK-293 cells (human embryonic kidney cells) were grown in Dulbecco’s modified eagle’s medium (DMEM) supplemented with 100 U/ml penicillin, 10% FBS, 100 mg/ml streptomycin and 50 mM glutamine. Furthermore, cells were harvested using trypsin and seeded in a 96-well cell culture plate for the MTT (3-(4,5-Dimethylthiazol-2-yl)-2,5-diphenyltetrazolium bromide) assay. The test compound was solubilized in cell culture grade DMSO. Once the cells in 96-well plates were 70% confluent, the compound was injected to the cells at five concentrations (0.01, 0.1, 1, 10, 100 *µ*M) at 37 °C for 48 h (made in DMEM supplemented with 1% antibiotic and 10% FBS). After 48 h, cells were washed with PBS and added with 100 *μ*L of fresh media in each well along with 10 *μ*L of MTT reagent (5 mg/mL) for another 4 h. After 4 h, the media was again removed and added 100 *μ*L cell culture grade DMSO to dissolve the formazan crystals formed by the reduction of MTT by live cells. The quantity of formed formazan crystal was determined as alterations in absorbance at 570 nm wavelength using ELISA plate reader (Bio-Tek). All tests were accomplished three times. The cell toxicity (%) was calculated using the following formula:

$$ \% \,{\rm{Cell}}\,{\rm{Toxicity}}=100-\frac{{\rm{OD}}\,{\rm{of}}\,{\rm{treated}}\,{\rm{well}}\,}{{\rm{OD}}\,{\rm{of}}\,{\rm{untreated}}\,{\rm{well}}}\times 100$$

### Procedure for DNA and BSA binding interactions

#### Sample preparation

The stock solution of compound **31** (10^−3^ M) was prepared in DMSO and diluted to required concentration using appropriate buffer solution. Calf thymus (CT)-DNA was dissolved in 10 mM Tris buffer of *p*H 7.4 having 1 mM EDTA at room temperature. The purity of DNA solution was determined by using the ratio of absorbance at 260 nm and 280 nm wavelength. Extinction coefficient 6600 M^−1^ cm^−1^ of single nucleotide at 260 nm was used to determine the concentration of the DNA solution. The stock solution of BSA (10^−3^ M) was prepared in distilled water and stored at 4 °C temperature.

#### Electronic absorption spectroscopy

In the case of DNA study, all absorption spectra of derivative **31** (20 *µ*M) were noted with incremental addition of CT-DNA (0–15 *µ*M) in phosphate buffer having *p*H 7.4. In case of BSA study, all absorption spectra were performed by taking BSA (10 *µ*M) and increasing concentration of compound **31** (0–8 *µ*M) in phosphate buffer having *p*H 7.4.

All spectra were recorded in the range of 200–800 nm using reference and sample cuvettes of 1 cm path length. Phosphate buffer was used for the corrections of baseline. Titration procedures were repeated until not any further change in spectrum was observed, demonstrating that saturation in the binding process has been achieved. Absorption data were then fit to the Benesi-Hildebrand equation (Eq. 1) to get binding constant (K_b_).

1 $$\frac{{{\rm{A}}}_{0}}{({\rm{A}}-{\rm{A}}0)}=\frac{{{\rm{\varepsilon }}}_{{\rm{f}}}}{({{\rm{\varepsilon }}}_{{\rm{b}}}-{}_{{\rm{f}}})}+\frac{{{\rm{\varepsilon }}}_{{\rm{f}}}}{({{\rm{\varepsilon }}}_{{\rm{b}}}-{}_{{\rm{f}}})\,{{\rm{K}}}_{{\rm{b}}}[{\rm{Analyte}}]}$$

A_o_ is the absorbance of derivative **31**/BSA in free form, whereas A is the absorbance of fully bound derivative **31**/BSA with the analyte (CT-DNA/derivative **31**). While ε_f_ and ε_b_ are denoting the molar extinction coefficients of derivative **31**/BSA in the absence and presence of the analyte, respectively. The ratio of intercept to the slope of plot A_o_/(A-A_o_) *vs*. 1/[analyte] gave the value K_b_.

#### Fluorescence quenching studies

In the case of DNA study, fluorescence emission spectra of derivative **31** (5 *µ*M) were noted with incremental addition of CT-DNA (0–50 *µ*M) in phosphate buffer (*p*H 7.4) at room temperature upon excitation at 290 nm. In the case of BSA experiments, fluorescence emission spectra were performed for BSA (10 *µ*M) with increasing concentration of compound **31** (0–10 *µ*M) in phosphate buffer (*p*H 7.4) at room temperature upon excitation at 280 nm. The dissociation constant of compound **31** was estimated by titrating the complex of compound **31** (10 *µ*M) and BSA (10 *µ*M) with increasing concentration of ibuprofen (0–15 *µ*M), upon excitation at 280 nm. All emission spectra were recorded in the range of 200 to 800 nm.

Binding parameters K_SV_ and K_q_ were calculated using the Stern-Volmer equation (Eq. 2):

2 $$\frac{{F}_{0}}{F}=1+{K}_{sv}[Analyte]=1+{K}_{q}{\tau }_{o}[Analyte]$$

F_o_ denotes the emission intensity of derivative **31**/BSA free from analyte (CT-DNA/ derivative **31**), whereas, F denotes the emission intensity of derivative **31**/BSA bound with analyte (CT-DNA/derivative **31**). The values of K_SV_ and K_q_ were determined from the plot of F_o_/F *vs*. [analyte].

The binding parameters K_bin_ and n were determined using Scatchard equation (Eq. 3):

3 $$\log \,\frac{{{\rm{F}}}_{0}-{\rm{F}}}{{\rm{F}}}={{\rm{logK}}}_{{\rm{bin}}}+{\rm{nlog}}[{\rm{analyte}}]$$

F_o_ and F denote similar factors as those of the above Stern-Volmer equation. The values of K_bin_ and n were determined from the plot of log (F_o_ − F)/F *vs*. log [analyte].

#### Competitive binding fluorescence measurements

Ethidium bromide (EtBr) displacement experiments were performed by incremental addition of compound **31** to complex of EtBr-DNA. The EtBr (3 *µ*M) and CT-DNA (30 *µ*M) were titrated with incremental addition of compound **31** (0–85 µM) in phosphate buffer (*p*H 7.4). The emission spectra of the EtBr-CT-DNA complex were noted in the range of 200 nm-800 nm using 520 nm as excitation wavelength. The quenching constant (K_q_) was calculated using the Stern-Volmer equation (Eq. 2). The apparent binding constant (K_app_) was obtained from known binding constant for ethidium bromide (K_EtBr_ = 1 × 10^7^ M^−1^) using the following equation (Eq. 4).

4 $${K}_{app}=\frac{{K}_{EtBr}[EtBr]}{{[compound]}_{50 \% }}$$

where [compound]_50%_ and [EtBr] are the concentrations of the compound at 50% quenching of DNA-bound ethidium bromide emission intensity and ethidium bromide (3 *µ*M), respectively.

#### Thermal denaturation studies

DNA melting studies were completed by measuring the absorption spectra at 260 nm of CT-DNA (8.5 *µ*M) and CT-DNA-derivative **31** complex (8.5 *µ*M:10 *µ*M) in phosphate buffer (*p*H 7.4) at varying temperature from 20 to 100 °C using spectrophotometer connected with Peltier.

#### Circular dichroism spectroscopy

The CD spectra of free CT-DNA (40 *µ*M) and CT-DNA-derivative **31** complex (40 *µ*M:1 *µ*M) were measured in 10 mM Tris-HCl buffer solution (*p*H 7.4). All CD spectra were measured from 220 to 400 nm. Tris-HCl buffer was used for the correction of baseline.

#### Fluorescence resonance energy-transfer studies

The energy transfer efficiency from BSA and compound **31** can be calculated using Eq. 5 ^27^:

5 $$E=1-\frac{F}{{F}_{{\rm{o}}}}=\frac{{R}_{{\rm{o}}}^{6}}{{R}_{{\rm{o}}}^{6}+{r}^{6}}$$

F and F_o_ are the BSA fluorescence intensities in the presence and absence of the derivative **31**, r is the distance among the acceptor and the donor, whereas R_o_ is the critical distance when the efficiency of energy transfer is 50%. R_o_ can be calculated using Eq. 6 ^28^:

6 $$\,{R}_{o}^{6}=8.8\times {10}^{-25}{k}^{2}{\eta }^{-4}\varPhi J$$

where *k*^2^ is the orientation factor of the dipole, *ɳ* is the refracted index of the medium, *Ф* the fluorescence quantum yield of the donor, and *J* is the overlap integral of the fluorescence emission spectrum of the donor with the absorption spectrum of the acceptor. The value of *J* can be calculated using Eq. 7 ^29^:

7 $$J=\frac{\varSigma \,F(\lambda )\varepsilon (\lambda ){\lambda }^{4}\varDelta \lambda }{\varSigma \,F(\lambda )\varDelta \lambda }$$

where F(λ) is fluorescence intensity of the donor i.e. BSA without acceptor and ε(λ) is molar absorption coefficient of the acceptor i.e. compound **31** at the wavelength λ, respectively. The values of *k*^2^ = 2/3, *ɳ* = 1.336 and *Ф* = 0.15 were used for the calculations.

### Physicochemical properties evaluation

Partition coefficients or experimental log P values of all synthesized compounds (**9–23**, **30** and **31**) were calculated using the shake-flask method. Log P values were determined using *n*-octanol and phosphate buffer (*p*H = 7.4) at 10:1 ratio of volumes. Stock solutions (10^−3^ M) of compounds were prepared in DMSO. Glass vials were used to make final solutions i.e. stock solution (250 *µ*L) and phosphate buffer (125 *µ*L). To record the absorbance, wavelength was selected based on λ_max_ of the respective compound. Initial absorbance (A_i_) for every compound was noted consuming the stock solution in the buffer phase. Now added *n*-octanol to every vial and shaken well using a mechanical shaker for 50 minutes. To get separation of both phases, glass vials were centrifuged for 35 min. at 2500 rpm. Then, the layer of octanol was separated from glass vial. The final absorbance (A_f_) of each compound was recorded by using the buffer layer. The values of P for compounds were determined by the following equation (Eq. 8):

8 $${\rm{P}}=\frac{{{\rm{A}}}_{{\rm{i}}-}{{\rm{A}}}_{{\rm{f}}}}{{{\rm{A}}}_{{\rm{f}}}}\times \frac{{{\rm{V}}}_{{\rm{w}}}}{{{\rm{V}}}_{{\rm{o}}}}$$

V_w_ represents the volume of aqueous phase, whereas V_o_ represents the volume of organic phase. Other pharmacokinetically significant properties of all synthesized compounds (**9-23**, **30** and **31**) like molecular weight, number of H-bond acceptors, number of H-bond donors and number of rotatable bonds were analyzed with the aid of molinspiration online property toolkit^30^. The percentage of absorptions (% ABS) for all compounds (**9-23**, **30** and **31**) were determined from the topological polar surface area (TPSA) by using the following Eq. 9 ^31^

9 $$ \% \,ABS=109-[0.345\times TPSA]$$

### Molecular docking

The AutoDock software package (vina) was used to execute the docking study of compound **31** with DNA (PdB: 1BNA). AutoDockTool (1.5.6rc3) was used to set up each ligand DNA interaction. Polar hydrogen atoms were added and water molecules were deleted. Gasteiger charges were calculated and nonpolar hydrogen atoms were merged to carbon atoms. To optimize the 3D structure of compound **31**, Gaussian 09 W program was used and saved it in pdf format. The ADT package (version 1.5.6rc3) was used to modify the partial charges of the pdf file of compound **31** and saved the resulting file into Pdbqt format. The size of a grid box 44 Å, 78 Å, 106 Å, indicating x, y and z directions was retained throughout the docking. The spacing of the grid was used to be 0.375 Å. All default settings were used to perform the docking.

### Statistical analysis

The experiments were performed in triplicates and the results were displayed as mean ± SD (Standard Deviations). The Microsoft Office Professional Plus 13 (excel) was used to plot the graph, and to calculate SD and other binding parameters.

## Supplementary information

Supplementary material.
