# Supplementary material for: Synthesis, cytotoxicity, pharmacokinetic profile, binding with DNA and BSA of new imidazo[1,2-a]pyrazine-benzo[d]imidazol-5-yl hybrids
Source: Sci Rep. 2020 Apr 16;10:6534. doi: 10.1038/s41598-020-63605-4 (PMC7162861; doi:10.1038/s41598-020-63605-4)
Supplement: Supplementary file 1 — Supplementary material. [file 41598_2020_63605_MOESM1_ESM.docx]

**Supporting Information**

**Synthesis, cytotoxicity, pharmacokinetic profile, binding with DNA and BSA of new imidazo[1,2-*a*]pyrazine-benzo[*d*]imidazol-5-yl hybrids**

Iqubal Singh, Vijay luxami and Kamaldeep Paul*

*School of Chemistry and Biochemistry, Thapar Institute of Engineering & Technology, Patiala- 147 004, India*

*E-mail:* [*kpaul@thapar.edu*](mailto:kpaul@thapar.edu)

**Table of Contents**

|  | **Contents** | **Page No** |
| --- | --- | --- |
|  | Experimental Details | S2-S8 |
| **Figures S1-S54** | ^1^H, ^13^C and Mass NMR spectra of compounds | S9-S35 |
| **Tables S1** | Anticancer activity of compounds at single dose concentration | S36-S37 |
| **Figure S55** | Benesi-Hildebrand plot of compound **31** for absorption spectra on incremental addition of CT-DNA | S37 |
| **Figure S56** | Stern–Volmer and Scatchard plot plots of compound **31** with CT-DNA | S37 |
| **Figure S57** | Stern–Volmer and Scatchard plot plots of compound **31** with EtBr-CT-DNA complex | S38 |
| **Figure S58** | Benesi-Hildebrand plots for interaction of BSA with compound **31** | S38 |
| **Figure S59** | Stern–Volmer and Scatchard plot plots for interaction of BSA with compound **31** | S38 |
| **Figure S60** | Electronic spectrum and Stern–Volmer plot for interaction of ibuprofen with compound **31**-BSA complex | S39 |
| **Table S2** | Binding energy of compound **31** based upon docking studies | S39 |

**Materials and methods**

All marketed chemicals were supplied from aldrich, spectrochem, etc. and were utilized as received without any purification. All determined melting points were uncorrected and recorded using the equiptronics digital melting point apparatus. Jeol ECS 400 spectrometer was used to record the ^1^H and ^13^C NMR spectra. Tetramethylsilane (TMS) and CDCl_3_ were used as an internal reference and solvent, respectively to record the NMR spectra. Mass spectra of derivatives were recorded by Water Micromass-Q-T of Micro. Thermo Scientific (Flash 2000) analyzer was used for elemental analysis. Derivatives were purified by column chromatography using 60-120 mess silica gel. Varian Cary Eclipse fluorescence spectrometer and Shimadzu UV-2600 machine were used to record emission and UV-Vis spectra, respectively. Applied Photophysics CD spectrophotometer was used to note the circular dichroism spectra.

*Procedure for synthesis of 1,4-dibromo-2-nitrobenzene (****2****)*: Compound **2** was synthesized as reported procedure, and was found as white solid (93% yield). Spectral data of compound **2** was found in agreement with data from the literature.^1^

*Procedure for synthesis of 4-bromo-N-cyclohexyl-2-nitroaniline (****3****):* Compound **3** was synthesized as reported procedure, and was found as yellow solid (75% yield). Spectral data of the compound **3** was found in agreement with data from the literature.^2^

*General procedure for synthesis of N-cyclohexyl-2-nitro-4-(4,4,5,5-tetramethyl-1,3,2-dioxaborolan-2-yl)aniline (****4****)*: 4-Bromo-*N*-cyclohexyl-2-nitroaniline (**3**) (2 gm, 6.68 mmol) was added to the oven-dried RBF (100 ml) equipped with a stir magnetic bar. Bis(pinacolato)diboron (2.03 gm, 8.01 mmol) was added to the RBF followed by the addition of potassium acetate (0.98 gm, 10.02 mmol). Then combined Pd(PPh_3_)_2_Cl_2_ (1.0 mol%) to RBF and added 20 ml of 1,4*-*dioxane. Under refluxing conditions, reaction was stirred for 10 h until full consumption of aryl halide. The solvent was evaporated and extraction was done with chloroform and water. The pure product was obtained by column chromatography using hexane and ethyl acetate as eluent. Reddish yellow solid was obtained in 82% isolated yield (1.89 gm); mp 125-127 ^o^C; ^1^H NMR (CDCl_3_, 400 MHz): δ (ppm) 8.60 (d, *J* = 1.44 Hz, 1H), 8.26 (d, *J* = 7.04 Hz, 1H), 7.72 (dd, *J* = 8.68, 0.96 Hz), 6.80 (d, *J* = 8.72 Hz, 1H), 3.55-3.48 (m, 1H), 2.06-2.00 (m, 2H), 1.79-1.73 (m, 2H), 1.64-1.60 (m, 1H), 1.45-1.20 (m, 17H); ^13^C NMR (CDCl_3_, 100 MHz): δ (ppm) 146.34, 141.47, 134.75, 131.58, 113.40, 84.67, 83.92, 51.00, 32.69, 25.62, 24.90, 24.57; MS (ESI): m/z 347.2 (M^+^+1); Anal Calcd for C_18_H_27_BN_2_O_4_: C, 62.44; H, 7.86; N, 8.09; found C, 62.40; H, 7.65; N, 8.23.

*General procedure for synthesis of 4-(6-bromoimidazo[1,2-a]pyrazin-8-yl)-N-cyclohexyl-2-nitroaniline (****6****)*: An oven dried RBF equipped with magnetic stir bar was charged with 3,6-dibromoimidazo[1,2-*a*]pyrazine (**5**) (2 g, 7.22 mmol) followed by addition of *N*-cyclohexyl-2-nitro-4-(4,4,5,5-tetramethyl-1,3,2-dioxaborolan-2-yl)aniline (**4**) (2.49 g, 7.22 mmol) in acetonitrile:water (9:1). Then added potassium carbonate (1.0 g, 7.22 mmol) and tetrakis(triphenylphosphine)palladium(0) (5 mol%) under inert atmosphere. The reaction was stirred under refluxing condition with continuous nitrogen supply. After completion, crude product was obtained by extraction with chloroform. Pure product was obtained by column chromatography using hexane : ethyl acetate (8:2) as eluents. Reddish solid was obtained in 73% isolated yield (2.18 gm); mp 158-160 ^o^C; ^1^H NMR (CDCl_3_, 400 MHz): δ (ppm) 9.84 (d, *J* = 2.16 Hz, 1H), 8.76 (dd, *J* = 9.20, 2.08 Hz, 1H), 8.40 (d, *J* = 7.36 Hz, 1H), 8.11 (s, 1H), 7.77 (d, *J* = 0.60 Hz, 1H), 7.65 (d, *J* = 0.56 Hz, 1H), 6.94 (d, *J* = 9.36 Hz, 1H), 3.58-3.56 (m, 1H), 2.07-2.05 (m, 2H), 1.81-1.75 (m, 2H), 1.66-1.63 (m, 1H), 1.48-1.28 (m, 5H ); ^13^C NMR (CDCl_3_, 100 MHz): δ (ppm) 146.67, 145.94, 138.08, 136.47, 135.87, 131.50, 129.87, 122.54, 121.75, 117.35, 114.09, 114.05, 51.34, 32.72, 25.59, 24.55.

*General procedure for synthesis of 4-(6-bromoimidazo[1,2-a]pyrazin-8-yl)-N^1^-cyclohexylbenzene-1,2-diamine (****7****)*: 4-(6-Bromoimidazo[1,2-*a*]pyrazin-8-yl)-*N*-cyclohexyl-2-nitroaniline **(6)** (2 gm, 4.80 mmol) was added to the oven-dried RBF (100 ml) followed by addition of sodium dithionite (4.18 gm, 24.03 mmol). Then added 20 ml of tetrahydrofuran : water (3:2) mixture followed by the addition of ammonia solution (6 ml). The reaction was stirred at room temperature for 1h. The crude brown product obtained by extraction with chloroform and was further used without purification.

*General procedure for synthesis of 6-bromo-8-(1-cyclohexyl-1H-benzo[d]imidazol-5-yl)imidazo[1,2-a]pyrazine (****8****)*: An oven dried RBF equipped with magnetic stir bar was charged with 4-(6-bromoimidazo[1,2-*a*]pyrazin-8-yl)-*N*^1^-cyclohexylbenzene-1,2-diamine (**7**) (1 gm, 2.59 mmol) in acetic acid. Triethylorthoformate (0.385 gm, 2.59 mmol) was added to the RBF and reaction was stirred at room temperature for 10 min. Crude product was obtained by extraction with chloroform after addition of sodium bicarbonate to basify the reaction. Pure product was obtained by column chromatography adopting hexane : ethyl acetate (7:3) as eluents. Light brown solid was obtained in 75% isolated yield (0.76 gm); mp 175-179 ^o^C; ^1^H NMR (CDCl_3_, 400 MHz): δ (ppm) 9.25 (s, 1H), 8.64 (dd, *J* = 8.68, 1.36 Hz, 1H), 8.14 (s, 1H), 8.05 (s, 1H), 7.78 (d, *J* = 0.68 Hz, 1H), 7.65 (s, 1H), 7.49 (d, *J* = 8.68 Hz, 1H), 4.22-4.14 (m, 1H), 2.20 (d, *J* = 11.52 Hz, 2H), 1.95 (d, *J* = 13.60 Hz, 2H), 1.81-1.71 (m, 3H), 1.53-1.38 (m, 2H), 1.33-1.21 (m, 1H); ^13^C NMR (CDCl_3_, 100 MHz): δ (ppm) 149.86, 143.73, 141.72, 138.72, 135.98, 135.47, 129.24, 124.60, 123.13, 122.69, 117.59, 114.07, 110.00, 55.69, 33.34, 25.71, 25.42; MS (ESI): m/z 396.3 (M^+^+1); Anal Calcd for C_19_H_18_BrN_5_: C, 57.59; H, 4.58; N, 17.67; found C, 57.49; H, 4.56; N, 17.53.

*Procedure for synthesis of 6-aryl-8-(1-cyclohexyl-1H-benzo[d]imidazol-6-yl)imidazo[1,2-a]pyrazine (****9-23****)*: Procedure for synthesis of 6-aryl-8-(1-cyclohexyl-1*H*-benzo[*d*]imidazol-6-yl)imidazo[1,2-*a*]pyrazine (**9-23**) is similar as that for the synthesis of 4-(6-bromoimidazo[1,2-*a*]pyrazin-8-yl)-*N*-cyclohexyl-2-nitroaniline (**6**) using 6-bromo-8-(1-cyclohexyl-1*H*-benzo[*d*]imidazol-5-yl)imidazo[1,2-*a*]pyrazine **(8)** and arylboronic acid.

*8-(1-Cyclohexyl-1H-benzo[d]imidazol-5-yl)-6-phenylimidazo[1,2-a]pyrazine (****9****)*: Light green solid; 126.53 mg, 85% yield; mp 195-198 ^o^C; ^1^H NMR (CDCl_3_, 400 MHz): δ (ppm) 9.33 (d, *J* = 1.12 Hz, 1H), 8.92 (dd, *J* = 8.68, 1.44 Hz, 1H), 8.43 (s, 1H), 8.10 (d, *J* = 5.60 Hz, 3H), 7.85 (d, *J* = 0.64 Hz, 1H), 7.76 (d, *J* = 0.64 Hz, 1H), 7.58 (d, *J* = 8.60 Hz, 1H), 7.52 (t, *J* = 7.20 Hz, 2H), 7.43 (t, *J* = 7.32 Hz, 1H), 4.29-4.21 (m, 1H), 2.28 (d, *J* = 13.56 Hz, 2H), 2.01 (d, *J* = 13.76 Hz, 2H), 1.88-1.79 (m, 3H), 1.56-1.49 (m, 2H), 1.41-1.34 (m, 1H); ^13^C NMR (CDCl_3_, 100 MHz): δ (ppm) 149.28, 141.50, 139.00, 138.85, 136.91, 135.23, 130.90, 128.95, 128.70, 126.33, 124.84, 122.38, 114.37, 113.56, 109.87, 55.78, 33.36, 25.75, 25.47; MS (ESI): m/z 394.3 (M^+^+1); Anal. Calcd for C_25_H_23_N_5_: C, 76.31; H, 5.89; N, 17.80; found C, 76.21; H, 5.78; N, 17.83.

*8-(1-Cyclohexyl-1H-benzo[d]imidazol-5-yl)-6-(4-methoxyphenyl)imidazo[1,2-a]pyrazine (****10****)*: Light green solid; 121.76 mg, 76% yield; mp 196-199 ^o^C; ^1^H NMR (CDCl_3_, 400 MHz): δ (ppm) 9.28 (s, 1H), 8.89 (d, *J* = 8.52 Hz, 1H), 8.32 (s, 1H), 8.13 (s, 1H), 8.00 (d, *J* = 8.44 Hz, 2H), 7.80 (s, 1H), 7.71 (s, 1H), 7.56 (d, *J* = 8.60 Hz, 1H), 7.00 (d, *J* = 8.48 Hz, 2H), 4.26-4.20 (m, 1H), 3.85 (s, 3H), 2.25 (d, *J* = 11.24 Hz, 2H), 1.99 (d, *J* = 12.96 Hz, 2H), 1.82-1.76 (m, 3H), 1.56-1.46 (m, 2H), 1.40-1.26 (m, 1H); ^13^C NMR (CDCl_3_, 100 MHz): δ (ppm) 160.15, 148.98, 141.43, 138.85, 138.65, 135.08, 131.05, 129.36, 127.55, 124.91, 122.20, 114.29, 112.57, 109.86, 55.80, 55.49, 33.35, 25.75, 25.46; MS (ESI): m/z 424.3 (M^+^+1); Anal Calcd for C_26_H_25_N_5_O: C, 73.74; H, 5.95; N, 16.54; found C, 73.86; H, 5.65; N, 16.23.

*4-(8-(1-Cyclohexyl-1H-benzo[d]imidazol-5-yl)imidazo[1,2-a]pyrazin-6-yl)benzaldehyde (****11****)*: Light green solid; 125.97 mg, 79% yield; mp 201-203 ^o^C; ^1^H NMR (CDCl_3_, 400 MHz): δ (ppm) 10.06 (s, 1H), 9.31 (d, *J* = 1.04 Hz, 1H), 8.91 (dd, *J* = 8.64, 1.40 Hz, 1H), 8.51 (s, 1H), 8.26 (d, *J* = 8.28 Hz, 2H), 8.08 (s, 1H), 7.98 (d, *J* = 8.28 Hz, 2H), 7.86 (s, 1H), 7.77 (s, 1H), 7.58 (d, *J* = 8.68 Hz, 1H), 4.29-4.21 (m, 1H), 2.28 (d, *J* = 11.20 Hz, 2H), 2.02 (d, *J* = 13.64 Hz, 2H), 1.88-1.78 (m, 3H), 1.60-1.48 (m, 2H), 1.39-1.27 (m, 1H); ^13^C NMR (CDCl_3_, 100 MHz): δ (ppm) 192.02, 149.49, 143.98, 142.55, 141.66, 138.95, 137.09, 136.08, 135.55, 135.29, 130.37, 130.32, 126.58, 124.72, 122.56, 114.79, 114.79, 109.87, 55.73, 33.37, 25.75, 25.47; MS (ESI): m/z 422.5 (M^+^+1); Anal Calcd for C_26_H_23_N_5_: C, 74.09; H, 5.50; N, 16.62; found C, 74.32; H, 5.62; N, 16.42.

*8-(1-Cyclohexyl-1H-benzo[d]imidazol-5-yl)-6-(naphthalen-1-yl)imidazo[1,2-a]pyrazine (****12****)*: Light green solid; 134.24 mg, 80% yield; mp 199-202 ^o^C; ^1^H NMR (CDCl_3_, 400 MHz): δ (ppm) 9.32 (d, *J* = 0.72 Hz, 1H), 8.79 (dd, *J* = 8.64, 1.32 Hz, 1H), 8.29-8.27 (m, 1H), 8.22 (s, 1H), 8.03 (s, 1H), 7.93 (d, *J* = 7.04 Hz, 2H), 7.88 (s, 1H), 7.75 (s, 1H), 7.67 (t, *J* = 6.60 Hz, 1H), 7.55-7.48 (m, 4H), 4.22-4.16 (m, 1H), 2.22 (d, *J* = 11.48 Hz, 2H), 1.97 (d, *J* = 13.44 Hz, 2H), 1.83-1.73 (m, 3H), 1.55-1.44 (m, 2H), 1.35-1.27 (m, 1H); ^13^C NMR (CDCl_3_, 100 MHz): δ (ppm) 149.35, 143.96, 141.48, 140.26, 138.76, 135.32, 135.27, 135.11, 134.11, 131.82, 130.59, 129.28, 128.48, 127.76, 126.67, 126.12, 125.93, 125.37, 124.66, 122.80, 117.21, 114.25, 109.86, 55.63, 33.34, 25.73, 25.46; MS (ESI): m/z 444.5 (M^+^+1); Anal Calcd for C_29_H_25_N_5_: C, 78.53; H, 5.68; N, 15.79; found C, 78.51; H, 5.75; N, 15.68.

*8-(1-Cyclohexyl-1H-benzo[d]imidazol-5-yl)-6-(4-fluorophenyl)imidazo[1,2-a]pyrazine (****13****)*: Light green solid; 126.09 mg, 81% yield; mp 187-189 ^o^C; ^1^H NMR (CDCl_3_, 400 MHz): δ (ppm) 9.30 (s, 1H), 8.90 (d, *J* = 8.60 Hz, 1H), 8.39 (s, 1H), 8.13 (s, 1H), 8.10-8.05 (m, 2H), 7.85 (s, 1H), 7.76 (s, 1H), 7.58 (d, *J* = 8.64 Hz, 1H), 7.20 (t, *J =* 8.52 Hz, 2H), 4.29-4.22 (m, 1H), 2.28 (d, *J* = 12.48 Hz, 2H), 2.02 (d, *J* = 13.64 Hz, 2H), 1.88-1.78 (m, 3H), 1.59-1.49 (m, 2H), 1.42-1.36 (m, 1H); ^13^C NMR (CDCl_3_, 100 MHz): δ (ppm) 149.26, 138.88, 137.96, 135.31, 132.98, 130.76, 128.10, 128.02, 124.83, 122.36, 115.97, 115.75, 114.39, 113.23, 109.87, 55.80, 33.36, 25.75, 25.46; MS (ESI): m/z 412.3 (M^+^+1); Anal Calcd for C_25_H_22_FN_5_: C, 72.97; H, 5.39; N, 17.02; found C, 72.85; H, 5.20; N, 17.01.

*8-(1-Cyclohexyl-1H-benzo[d]imidazol-5-yl)-6-(thiophen-3-yl)imidazo[1,2-a]pyrazine (****14****)*: Light green solid; 126.94 mg, 84% yield; mp 185-188 ^o^C; ^1^H NMR (CDCl_3_, 400 MHz): δ (ppm) 9.28 (s, 1H), 8.90 (d, *J* = 8.64 Hz, 1H), 8.34 (s, 1H), 8.23 (s, 1H), 8.04 (d, *J* = 2.16 Hz, 1H), 7.83 (s, 1H), 7.74 (s, 1H), 7.62 (d, *J* = 4.52 Hz, 2H), 7.45 (dd, *J =* 4.76, 3.00 Hz, 1H), 4.32-4.26 (m, 1H), 2.29 (d, *J* = 12.16 Hz, 2H), 2.03 (d, *J* = 13.60 Hz, 2H), 1.89-1.80 (m, 3H), 1.64-1.46 (m, 2H), 1.42-1.28 (m, 1H); ^13^C NMR (CDCl_3_, 100 MHz): δ (ppm) 149.29, 139.06, 138.88, 135.61, 135.19, 131.04, 126.79, 125.08, 123.01, 122.15, 114.33, 113.09, 110.01, 55.97, 33.36, 25.74, 25.44; MS (ESI): m/z 400.2 (M^+^+1); Anal Calcd for C_23_H_21_N_5_S: C, 69.15; H, 5.30; N, 17.53; S, 8.02; found C, 69.26; H, 5.34; N, 17.51; S, 8.13.

*8-(1-Cyclohexyl-1H-benzo[d]imidazol-5-yl)-6-(m-tolyl)imidazo[1,2-a]pyrazine (****15****)*: Light green solid; 117.16 mg, 76% yield; mp 183-186 ^o^C; ^1^H NMR (CDCl_3_, 400 MHz): δ (ppm) 9.32 (d, *J* = 0.68 Hz, 1H), 8.93 (dd, *J* = 8.64, 1.28 Hz, 1H), 8.38 (s, 1H), 8.06 (s, 1H), 7.93 (s, 1H), 7.84 (d, *J* = 7.44 Hz, 2H), 7.73 (d, *J* = 0.64 Hz, 1H), 7.57 (d, *J* = 8.64 Hz, 1H), 7.38 (t, *J* = 7.60 Hz, 1H), 7.22 (d, *J* = 7.56 Hz, 1H), 4.27-4.19 (m, 1H), 2.45 (s, 3H), 2.26 (d, *J* = 11.48 Hz, 2H), 1.99 (d, *J* = 13.56 Hz, 2H), 1.86-1.76 (m, 3H), 1.58-1.46 (m, 2H), 1.37-1.25 (m, 1H); ^13^C NMR (CDCl_3_, 100 MHz): δ (ppm) 149.23, 144.08, 141.51, 139.01, 138.84, 138.62, 136.73, 135.14, 130.78, 129.42, 128.78, 127.07, 124.78, 123.27, 122.49, 114.33, 113.52, 109.75, 55.67, 33.35, 25.75, 25.48, 21.75; MS (ESI): m/z 408.6 (M^+^+1); Anal Calcd for C_26_H_25_N_5_: C, 76.63; H, 6.18; N, 17.19; found C, 76.51; H, 6.10; N, 17.37.

*8-(1-Cyclohexyl-1H-benzo[d]imidazol-5-yl)-6-(4-ethylphenyl)imidazo[1,2-a]pyrazine (****16****)*: Light green solid; 132.35 mg, 83% yield; mp 182-185 ^o^C; ^1^H NMR (CDCl_3_, 400 MHz): δ (ppm) 9.33 (s, 1H), 8.93 (dd, *J* = 8.64, 1.20 Hz, 1H), 8.37 (s, 1H), 8.06 (s, 1H), 8.01 (d, *J* = 8.16 Hz, 2H), 7.82 (s, 1H), 7.73 (s, 1H), 7.57 (d, *J* = 8.68 Hz, 1H), 7.33 (d, *J* = 8.08 Hz, 2H), 4.27-4.19 (m, 1H), 2.74 (q, *J* = 7.64 Hz, 2H), 2.26 (d, *J* = 11.36 Hz, 2H), 2.00 (d, *J* = 13.56 Hz, 2H), 1.86-1.76 (m, 3H), 1.58-1.47 (m, 2H), 1.38-1.23 (m, 4H); ^13^C NMR (CDCl_3_, 100 MHz): δ (ppm) 149.16, 144.95, 144.07, 141.50, 138.99, 138.84, 135.09, 134.26, 130.81, 128.45, 126.27, 124.75, 122.51, 114.28, 113.09, 109.73, 55.66, 33.36, 28.75, 25.76, 25.48, 15.71; MS (ESI): m/z 422.5 (M^+^+1); Anal Calcd for C_27_H_27_N_5_: C, 76.93; H, 6.46; N, 16.61; found C, 76.72; H, 6.59; N, 16.50.

*1-(4-(8-(1-Cyclohexyl-1H-benzo[d]imidazol-5-yl)imidazo[1,2-a]pyrazin-6-yl)phenyl)ethan-1-one (****17****)*: Light green solid; 128.52 mg, 78% yield; mp 200-203 ^o^C; ^1^H NMR (CDCl_3_, 400 MHz): δ (ppm) 9.32 (s, 1H), 8.92 (dd, *J* = 8.60, 1.24 Hz, 1H), 8.52 (s, 1H), 8.20 (d, *J* = 8.36 Hz, 2H), 8.13 (s, 1H), 8.08 (d, *J* = 8.36 Hz, 2H), 7.87 (s, 1H), 7.79 (s, 1H), 7.59 (d, *J* = 8.68 Hz, 1H), 4.30-4.22 (m, 1H), 2.66 (s, 3H), 2.29 (d, *J* = 11.80 Hz, 2H), 2.02 (d, *J* = 13.68 Hz, 2H), 1.88-1.80 (m, 3H), 1.60-1.50 (m, 2H), 1.39-1.30 (m, 1H); ^13^C NMR (CDCl_3_, 100 MHz): δ (ppm) 197.89, 149.46, 143.64, 141.60, 141.22, 138.99, 137.39, 136.85, 135.52, 135.18, 130.60, 129.01, 126.22, 124.83, 122.44, 114.64, 114.54, 109.91, 55.80, 33.37, 26.86, 25.75, 25.46; MS (ESI): m/z 436.4 (M^+^+1); Anal Calcd for C_27_H_25_N_5_O: C, 74.46; H, 5.79; N, 16.08; found C, 74.77; H, 5.72; N, 16.01.

*6-(4-Chlorophenyl)-8-(1-cyclohexyl-1H-benzo[d]imidazol-5-yl)imidazo[1,2-a]pyrazine (****18****)*: Light green solid; 121.30 mg, 75% yield; mp 193-196 ^o^C; ^1^H NMR (CDCl_3_, 400 MHz): δ (ppm) 9.28 (s, 1H), 8.88 (d, *J* = 8.56 Hz, 1H), 8.39 (s, 1H), 8.06 (s, 1H), 8.02 (d, *J* = 8.40 Hz, 2H), 7.83 (s, 1H), 7.74 (s, 1H), 7.56 (d, *J* = 8.64 Hz, 1H), 7.45 (d, *J* = 8.44 Hz, 2H), 4.26-4.20 (m, 1H), 2.26 (d, *J* = 11.60 Hz, 2H), 1.99 (d, *J* = 12.48 Hz, 2H), 1.83-1.77 (m, 3H), 1.57-1.47 (m, 2H), 1.41-1.26 (m, 1H); ^13^C NMR (CDCl_3_, 100 MHz): δ (ppm) 149.43, 144.01, 141.59, 138.95, 137.68, 135.36, 135.23, 134.62, 130.55, 129.09, 127.52, 124.73, 122.54, 114.44, 113.44, 109.81, 55.72, 33.38, 25.77, 25.49; MS (ESI): m/z 428.4 (M^+^+1); Anal Calcd for C_25_H_22_ClN_5_: C, 70.17; H, 5.18; N, 16.37; found C, 70.25; H, 5.25; N, 16.34.

*6-(4-Bromophenyl)-8-(1-cyclohexyl-1H-benzo[d]imidazol-5-yl)imidazo[1,2-a]pyrazine (****19****)*: Light green solid; 141.24 mg, 79% yield; mp 195-198 ^o^C; ^1^H NMR (CDCl_3_, 400 MHz): δ (ppm) 9.30 (d, *J* = 0.76 Hz, 1H), 8.89 (dd, *J* = 8.56, 1.32 Hz, 1H), 8.40 (s, 1H), 8.09 (s, 1H), 7.96 (d, *J* = 8.56 Hz, 2H), 7.84 (d, *J* = 0.68 Hz, 1H), 7.74 (d, *J* = 0.68 Hz, 1H), 7.61 (d, *J* = 8.56 Hz, 2H), 7.57 (s, 1H), 4.28-4.20 (m, 1H), 2.27 (d, *J* = 9.96 Hz, 2H), 2.01 (d, *J* = 12.00 Hz, 2H), 1.87-1.77 (m, 3H), 1.59-1.49 (m, 2H), 1.44-1.31 (m, 1H); ^13^C NMR (CDCl_3_, 100 MHz): δ (ppm) 149.35, 143.91, 141.58, 138.92, 137.65, 135.76, 135.35, 135.19, 132.01, 130.56, 129.20, 128.70, 127.80, 127.36, 126.74, 124.75, 122.86, 122.49, 114.47, 113.47, 109.82, 55.73, 33.37, 25.76, 25.48; MS (ESI): m/z 472.1 (M^+^+1); Anal Calcd for C_25_H_22_BrN_5_: C, 63.57; H, 4.69; N, 14.83; found C, 63.51; H, 4.74; N, 14.88.

*8-(1-Cyclohexyl-1H-benzo[d]imidazol-5-yl)-6-(2-methoxyphenyl)imidazo[1,2-a]pyrazine (****20****)*: Light green solid; 128.18 mg, 80% yield; mp 195-198 ^o^C; ^1^H NMR (CDCl_3_, 400 MHz): δ (ppm) 9.29 (s, 1H), 8.91 (dd, *J* = 8.68, 1.08 Hz, 1H), 8.87 (s, 1H), 8.46 (dd, *J* = 7.80, 1.68 Hz, 1H), 8.06 (s, 1H), 7.83 (s, 1H), 7.74 (d, *J* =0.72 Hz, 1H), 7.57 (d, *J* = 8.64 Hz, 1H), 7.39-7.34 (m, 1H), 7.18 (t, *J* = 7.40 Hz, 1H), 7.03 (d, *J* = 8.20 Hz, 1H), 4.27-4.20 (m, 1H), 3.96 (s, 3H), 2.26 (d, *J* = 11.40 Hz, 2H), 2.00 (d, *J* = 13.60 Hz, 2H), 1.86-1.76 (m, 3H), 1.57-1.48 (m, 2H), 1.37-1.30 (m, 1H); ^13^C NMR (CDCl_3_, 100 MHz): δ (ppm) 156.88, 148.81, 144.06, 141.43, 138.79, 135.15, 134.97, 131.09, 130.99, 129.53, 125.30, 124.70, 122.32, 121.32, 118.32, 114.35, 111.31, 109.70, 77.48, 55.74, 55.66, 33.37, 25.76, 25.49; MS (ESI): m/z 424.3 (M^+^+1); Anal Calcd for C_26_H_25_N_5_O: C, 73.74; H, 5.95; N, 16.54; found C, 73.80; H, 5.90; N, 16.44.

*8-(1-Cyclohexyl-1H-benzo[d]imidazol-5-yl)-6-(2-fluorophenyl)imidazo[1,2-a]pyrazine (****21****)*: Light green solid; 133.88 mg, 86% yield; mp 190-193 ^o^C; ^1^H NMR (CDCl_3_, 400 MHz): δ (ppm) 9.28 (s, 1H), 8.88 (d, *J* = 8.60 Hz, 1H), 8.37 (s, 1H), 8.11-8.03 (m, 3H), 7.83 (s, 1H), 7.74 (s, 1H), 7.56 (d, *J* = 8.64 Hz, 1H), 7.20-7.14 (m, 2H), 4.27-4.20 (m, 1H), 2.26 (d, *J* = 12.48 Hz, 2H), 2.00 (d, *J* = 13.64 Hz, 2H), 1.86-1.77 (m, 3H), 1.57-1.47 (m, 2H), 1.40-1.30 (m, 1H); ^13^C NMR (CDCl_3_, 100 MHz): δ (ppm) 149.26, 141.54, 138.88, 137.96, 135.31, 132.98, 130.76, 128.10, 128.02, 124.83, 122.36, 115.97, 115.75, 114.39, 113.23, 109.87, 55.80, 33.36, 25.75, 25.46; MS (ESI): m/z 412.3 (M^+^+1); Anal Calcd for C_25_H_22_FN_5_: C, 72.97; H, 5.39; N, 17.02; found C, 72.95; H, 5.43; N, 17.08.

*8-(1-Cyclohexyl-1H-benzo[d]imidazol-5-yl)-6-(thiophen-2-yl)imidazo[1,2-a]pyrazine (****22****)*: Light green solid; 120.90 mg, 80% yield; mp 187-190 ^o^C; ^1^H NMR (CDCl_3_, 400 MHz): δ (ppm) 9.32 (s, 1H), 8.89 (dd, *J* = 8.64, 0.96 Hz, 1H), 8.37 (s, 1H), 8.08 (s, 1H), 7.84 (s, 1H), 7.74 (s, 1H), 7.60-7.56 (m, 2H), 7.40 (d, *J* = 5.00 Hz, 1H), 7.15 (q, *J* = 4.92 Hz, 1H), 4.29-4.22 (m, 1H), 2.28 (d, *J* = 10.24 Hz, 2H), 2.02 (d, *J* = 13.28 Hz, 2H), 1.89-1.78 (m, 3H), 1.60-1.49 (m, 2H), 1.40-1.27 (m, 1H); ^13^C NMR (CDCl_3_, 100 MHz): δ (ppm) 149.29, 139.05, 138.87, 135.60, 135.18, 131.03, 126.78, 125.08, 123.00, 122.15, 114.32, 113.08, 110.00, 55.97, 33.36, 25.74, 25.43; MS (ESI): m/z 400.2 (M^+^+1); Anal Calcd for C_23_H_21_N_5_S: C, 69.15; H, 5.30; N, 17.53; S, 8.02; found C, 69.17; H, 5.26; N, 17.58; S, 8.08.

*8-(1-Cyclohexyl-1H-benzo[d]imidazol-5-yl)-6-(3-(trifluoromethyl)phenyl)imidazo[1,2-a]pyrazine (****23****)*: Light green solid; 141.44 mg, 81% yield; mp 196-199 ^o^C; ^1^H NMR (CDCl_3_, 400 MHz): δ (ppm) 9.33 (s, 1H), 8.89 (dd, *J* = 8.60, 1.04 Hz, 1H), 8.50 (s, 1H), 8.32 (d, *J* = 6.36 Hz, 2H), 8.08 (s, 1H), 7.88 (s, 1H), 7.80 (s, 1H), 7.68-7.63 (m, 2H), 7.60 (d, *J =* 8.68 Hz, 1H), 4.30-4.22 (m, 1H), 2.29 (d, *J* = 11.52 Hz, 2H), 2.02 (d, *J* = 13.60 Hz, 2H), 1.89-1.79 (m, 3H), 1.60-1.50 (m, 2H), 1.40-1.28 (m, 1H); ^13^C NMR (CDCl_3_, 100 MHz): δ (ppm) 149.73, 144.07, 141.62, 139.03, 137.73, 137.39, 135.55, 135.31, 131.49, 130.39, 129.62, 129.42, 125.26, 124.69, 122.90, 122.71, 114.59, 113.99, 109.89, 55.73, 33.39, 25.77, 25.48; MS (ESI): m/z 462.4 (M^+^+1); Anal Calcd for C_26_H_22_F_3_N_5_: C, 67.67; H, 4.81; N, 15.18; found C, 67.73; H, 4.98; N, 15.35.

*2,4-Dibromo-1-nitrobenzene (****25****)*: Compound **25** was synthesized as reported procedure, and was found as white solid (96% yield). Spectral data of the compound **25** was found in agreement with data from the literature.^2^

*5-Bromo-N-cyclohexyl-2-nitroaniline (****26****)*: Compound **26** was synthesized as reported procedure, and was found as yellow solid (74% yield). Spectral data of the compound **26** was found in agreement with data from the literature.^2^

*4-bromo-N^1^-cyclohexylbenzene-1,2-diamine (****27a****)*: Compound **27a** was synthesized as reported procedure.^2^

*5-Bromo-N^1^-cyclohexylbenzene-1,2-diamine (****27b****)*: Compound **27b** was synthesized as reported procedure.^2^

*5-Bromo-1-cyclohexyl-1H-benzo[d]imidazole (****28a****)*: Compound **28a** was synthesized as reported procedure.^2^

*6-Bromo-1-cyclohexyl-1H-benzo[d]imidazole (****28b****)*: Compound **28b** was synthesized as reported procedure.^2^

*1-Cyclohexyl-5-(4,4,5,5-tetramethyl-1,3,2-dioxaborolan-2-yl)-1H-benzo[d]imidazole (****29a****)*: Compound **29a** was synthesized as reported procedure.^2^

*1-Cyclohexyl-6-(4,4,5,5-tetramethyl-1,3,2-dioxaborolan-2-yl)-1H-benzo[d]imidazole (****29b****)*: Compound **29b** was synthesized as reported procedure.^2^

*Procedure for synthesis of 6,8-(bisbenzimidazole)imidazo[1,2-a]pyrazine (****30-31****)*: Procedure for synthesis of *6,8-(bisbenzimidazole)imidazo[1,2-a]pyrazine (****30-31****)* is similar as that for the synthesis of 4-(6-bromoimidazo[1,2-*a*]pyrazin-8-yl)-*N*-cyclohexyl-2-nitroaniline (**6**) using 1-cyclohexyl-5-(4,4,5,5-tetramethyl-1,3,2-dioxaborolan-2-yl)-1*H*-benzo[*d*]imidazole (**29a**) or 1-cyclohexyl-6-(4,4,5,5-tetramethyl-1,3,2-dioxaborolan-2-yl)-1*H*-benzo[*d*]imidazole (**29b**) and *6-bromo-8-(1-cyclohexyl-1H-benzo[d]imidazol-5-yl)imidazo[1,2-a]pyrazine* (**8**).

*6,8-Bis(1-cyclohexyl-1H-benzo[d]imidazol-5-yl)imidazo[1,2-a]pyrazine (****30****)*: Light brown solid; 146.30 mg, 75% yield; mp 205-209 ^o^C; ^1^H NMR (CDCl_3_, 400 MHz): δ (ppm) 8.44 (s, 1H), 8.24 (d, *J* = 1.24 Hz, 1H), 8.05 (s, 2H), 8.03 (s, 1H), 7.94 (dd, *J* = 8.56, 1.52 Hz, 1H), 7.82 (s, 2H), 7.61 (dd, *J* = 8.40, 1.52 Hz, 1H), 7.52 (d, *J* = 3.64 Hz, 1H), 7.50 (d, *J* = 3.52 Hz, 1H), 4.26-4.18 (m, 2H), 2.26 (d, *J* = 12.60 Hz, 4H), 2.00 (d, *J* = 13.24 Hz, 4H), 1.87-1.75 (m, 6H), 1.57-1.47 (m, 4H), 1.39-1.27 (m, 2H); ^13^C NMR (CDCl_3_, 100 MHz): δ (ppm) 144.38, 144.23, 142.69, 141.65, 141.05, 139.65, 137.29, 136.51, 136.06, 134.21, 132.63, 129.53, 122.89, 121.73, 118.98, 117.94, 116.09, 114.35, 110.75, 110.38, 55.73, 55.69, 33.38, 25.78, 25.74, 25.47, 25.42; MS (ESI): m/z 516.6 (M^+^+1); Anal Calcd for C_32_H_33_N_7_: C, 74.54; H, 6.45; N, 19.01; found C, 74.92; H, 6.25; N, 19.11.

*8-(1-Cyclohexyl-1H-benzo[d]imidazol-5-yl)-6-(1-cyclohexyl-1H-benzo[d]imidazol-6-yl)imidazo[1,2-a]pyrazine (****31****)*: Light brown solid; 158.0 mg, 81% yield; mp 203-206 ^o^C; ^1^H NMR (CDCl_3_, 400 MHz): δ (ppm) 9.25 (d, *J* = 1.20 Hz, 1H), 8.86 (dd, *J* = 8.60, 1.40 Hz, 1H), 8.46 (s, 1H), 8.22 (s, 1H), 8.08 (d, *J* = 7.84 Hz, 2H), 7.87-7.82 (m, 3H), 7.77 (d, *J* = 0.60 Hz, 1H), 7.58 (d, *J* = 8.68 Hz, 1H), 4.38-4.30 (m, 1H), 4.28-4.20 (m, 1H), 2.26 (s (b), 4H), 2.02-1.96 (m, 4H), 1.87-1.74 (m, 6H), 1.62-1.47 (m, 4H, 2), 1.38-1.26 (m, 2H); ^13^C NMR (CDCl_3_, 100 MHz): δ (ppm) 149.60, 144.15, 144.00, 141.56, 141.39, 139.56, 139.01, 135.28, 135.13, 134.21, 131.89, 130.80, 124.84, 122.52, 120.51, 114.33, 113.68, 109.81, 108.56, 55.72, 55.21, 33.72, 33.39, 25.77, 25.71, 25.49; MS (ESI): m/z 516.6 (M^+^+1); Anal Calcd for C_32_H_33_N_7_: C, 74.54; H, 6.45; N, 19.01; found C, 74.48; H, 6.39; N, 19.26.

**Figure S1.** ^1^H NMR spectrum of *N*-cyclohexyl-2-nitro-4-(4,4,5,5-tetramethyl-1,3,2-dioxaborolan-2-yl)aniline (**4**).

**Figure S2.** ^13^C NMR spectrum of *N*-cyclohexyl-2-nitro-4-(4,4,5,5-tetramethyl-1,3,2-dioxaborolan-2-yl)aniline (**4**).

**Figure S3.** ^1^H NMR spectrum of 4-(6-bromoimidazo[1,2-*a*]pyrazin-8-yl)-*N*-cyclohexyl-2-nitroaniline (**6**).

**Figure S4.** ^13^C NMR spectrum of 4-(6-bromoimidazo[1,2-*a*]pyrazin-8-yl)-*N*-cyclohexyl-2-nitroaniline (**6**).

**Figure S5.** ^1^H NMR spectrum of 6-bromo-8-(1-cyclohexyl-1*H*-benzo[*d*]imidazol-5-yl)imidazo[1,2-*a*]pyrazine (**8**).

**Figure S6.** ^13^C NMR spectrum of 6-bromo-8-(1-cyclohexyl-1*H*-benzo[*d*]imidazol-5-yl)imidazo[1,2-*a*]pyrazine (**8**).

**
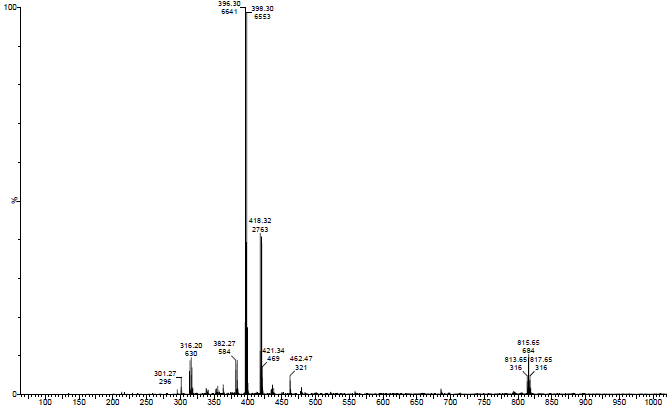
Figure S7.** Mass spectrum of 6-bromo-8-(1-cyclohexyl-1*H*-benzo[*d*]imidazol-5-yl)imidazo[1,2-*a*]pyrazine (**8**).

**Figure S8.** ^1^H NMR spectrum of 8-(1-cyclohexyl-1*H*-benzo[*d*]imidazol-5-yl)-6-phenylimidazo[1,2-*a*]pyrazine (**9**).

**Figure S9.** ^13^C NMR spectrum of 8-(1-cyclohexyl-1*H*-benzo[*d*]imidazol-5-yl)-6-phenylimidazo[1,2-*a*]pyrazine (**9**).


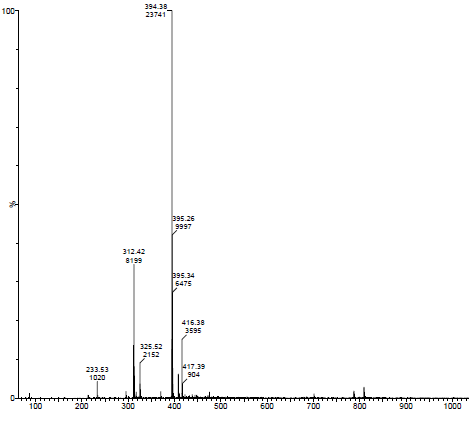

**Figure S10.** Mass spectrum of 8-(1-cyclohexyl-1*H*-benzo[*d*]imidazol-5-yl)-6-phenylimidazo[1,2-*a*]pyrazine (**9**).

**Figure S11.** ^1^H NMR spectrum of 8-(1-cyclohexyl-1*H*-benzo[*d*]imidazol-5-yl)-6-(4-methoxyphenyl)imidazo[1,2-*a*]pyrazine (**10**).

**Figure S12.** ^13^C NMR spectrum of 8-(1-cyclohexyl-1*H*-benzo[*d*]imidazol-5-yl)-6-(4-methoxyphenyl)imidazo[1,2-*a*]pyrazine (**10**).

**
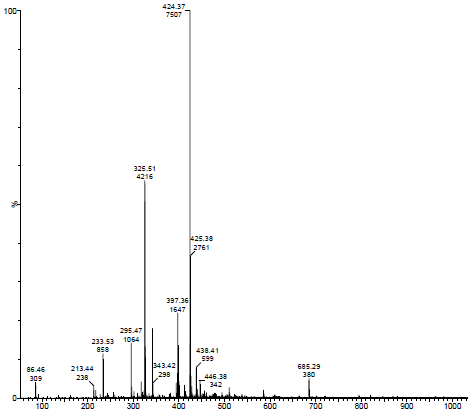
**

**Figure S13.** Mass spectrum of 8-(1-cyclohexyl-1*H*-benzo[*d*]imidazol-5-yl)-6-(4-methoxyphenyl)imidazo[1,2-*a*]pyrazine (**10**).

**Figure S14.** ^1^H NMR spectrum of 4-(8-(1-cyclohexyl-1*H*-benzo[*d*]imidazol-5-yl)imidazo[1,2-*a*]pyrazin-6-yl)benzaldehyde (**11**).

**Figure S15.** ^13^C NMR spectrum of 4-(8-(1-cyclohexyl-1*H*-benzo[*d*]imidazol-5-yl)imidazo[1,2-*a*]pyrazin-6-yl)benzaldehyde (**11**).

**
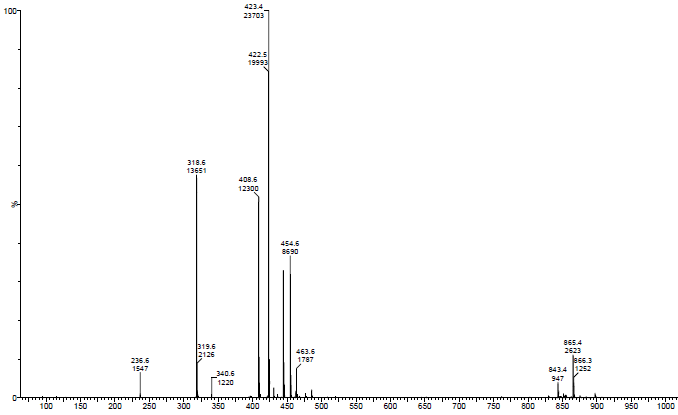
**

**Figure S16.** Mass spectrum of 4-(8-(1-cyclohexyl-1*H*-benzo[*d*]imidazol-5-yl)imidazo[1,2-*a*]pyrazin-6-yl)benzaldehyde (**11**).

**Figure S17.** ^1^H NMR spectrum of 8-(1-cyclohexyl-1*H*-benzo[*d*]imidazol-5-yl)-6-(naphthalen-1-yl)imidazo[1,2-*a*]pyrazine (**12**).

**Figure S18.** ^13^C NMR spectrum of 8-(1-cyclohexyl-1*H*-benzo[*d*]imidazol-5-yl)-6-(naphthalen-1-yl)imidazo[1,2-*a*]pyrazine (**12**).

**
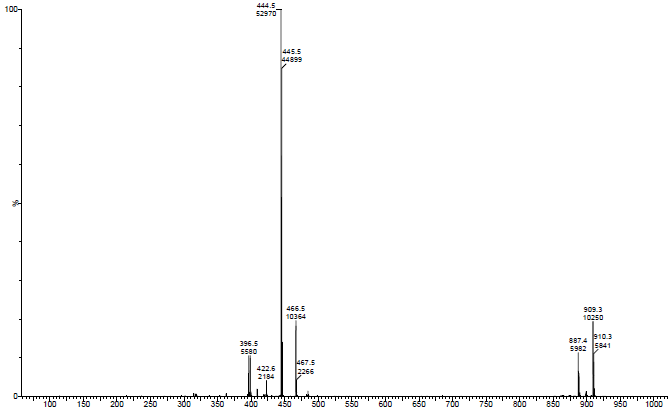
Figure S19.** Mass spectrum of 8-(1-cyclohexyl-1*H*-benzo[*d*]imidazol-5-yl)-6-(naphthalen-1-yl)imidazo[1,2-*a*]pyrazine (**12**).

**Figure S20.** ^1^H NMR spectrum of 8-(1-cyclohexyl-1*H*-benzo[*d*]imidazol-5-yl)-6-(4-fluorophenyl)imidazo[1,2-*a*]pyrazine (**13**).

**Figure S21.** ^13^C NMR spectrum of 8-(1-cyclohexyl-1*H*-benzo[*d*]imidazol-5-yl)-6-(4-fluorophenyl)imidazo[1,2-*a*]pyrazine (**13**).


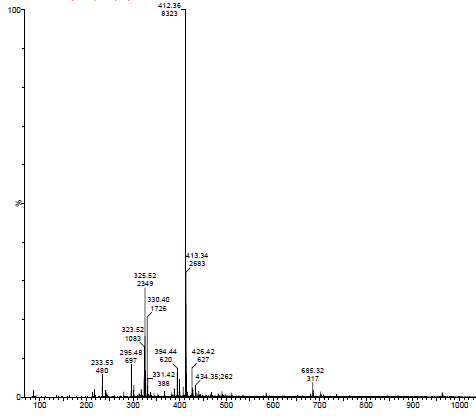

**Figure S22.** Mass spectrum of 8-(1-cyclohexyl-1*H*-benzo[*d*]imidazol-5-yl)-6-(4-fluorophenyl)imidazo[1,2-*a*]pyrazine (**13**).

**Figure S23.** ^1^H NMR spectrum of 8-(1-cyclohexyl-1*H*-benzo[*d*]imidazol-5-yl)-6-(thiophen-3-yl)imidazo[1,2-*a*]pyrazine (**14**).

**Figure S24.** ^13^C NMR spectrum of 8-(1-cyclohexyl-1*H*-benzo[*d*]imidazol-5-yl)-6-(thiophen-3-yl)imidazo[1,2-*a*]pyrazine (**14**).

**
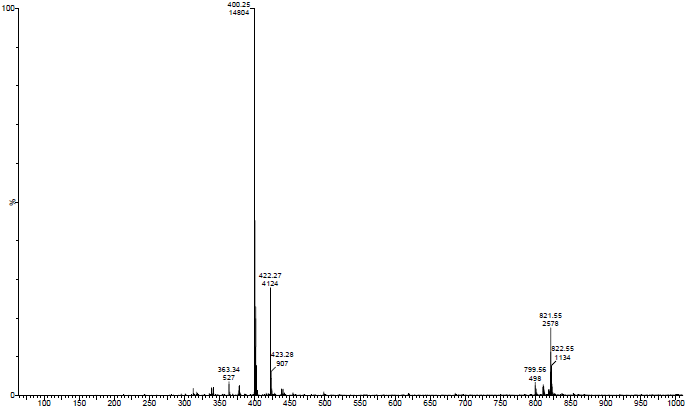
Figure S25.** Mass spectrum of 8-(1-cyclohexyl-1*H*-benzo[*d*]imidazol-5-yl)-6-(thiophen-3-yl)imidazo[1,2-*a*]pyrazine (**14**).

**Figure S26.** ^1^H NMR spectrum of 8-(1-cyclohexyl-1*H*-benzo[*d*]imidazol-5-yl)-6-(*m*-tolyl)imidazo[1,2-*a*]pyrazine (**15**).

**Figure S27.** ^13^C NMR spectrum of 8-(1-cyclohexyl-1*H*-benzo[*d*]imidazol-5-yl)-6-(*m*-tolyl)imidazo[1,2-*a*]pyrazine (**15**).

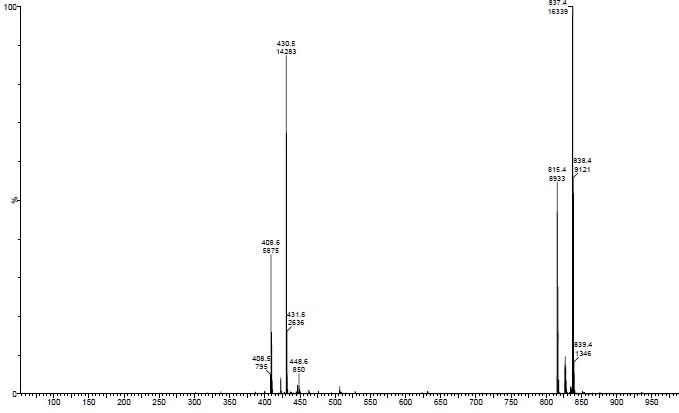


**Figure S28.** Mass spectrum of 8-(1-cyclohexyl-1*H*-benzo[*d*]imidazol-5-yl)-6-(*m*-tolyl)imidazo[1,2-*a*]pyrazine (**15**).

**Figure S29.** ^1^H NMR spectrum of 8-(1-cyclohexyl-1*H*-benzo[*d*]imidazol-5-yl)-6-(4-ethylphenyl)imidazo[1,2-*a*]pyrazine (**16**).

**Figure S30.** ^13^C NMR spectrum of 8-(1-cyclohexyl-1*H*-benzo[*d*]imidazol-5-yl)-6-(4-ethylphenyl)imidazo[1,2-*a*]pyrazine (**16**).

**
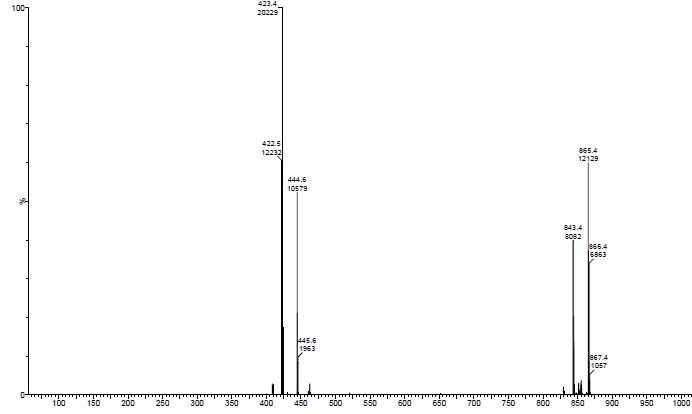
Figure S31.** Mass spectrum of 8-(1-cyclohexyl-1*H*-benzo[*d*]imidazol-5-yl)-6-(4-ethylphenyl)imidazo[1,2-*a*]pyrazine (**16**).

**Figure S32.** ^1^H NMR spectrum of 1-(4-(8-(1-cyclohexyl-1*H*-benzo[*d*]imidazol-5-yl)imidazo[1,2-*a*]pyrazin-6-yl)phenyl)ethan-1-one (**17**).

**Figure S33.** ^13^C NMR spectrum of 1-(4-(8-(1-cyclohexyl-1*H*-benzo[*d*]imidazol-5-yl)imidazo[1,2-*a*]pyrazin-6-yl)phenyl)ethan-1-one (**17**).

**
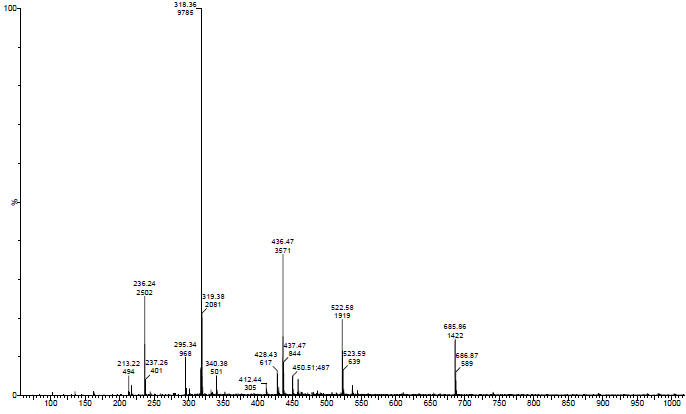
Figure S34.** Mass spectrum of 1-(4-(8-(1-cyclohexyl-1*H*-benzo[*d*]imidazol-5-yl)imidazo[1,2-*a*]pyrazin-6-yl)phenyl)ethan-1-one (**17**).

**Figure S35.** ^1^H NMR spectrum of 6-(4-chlorophenyl)-8-(1-cyclohexyl-1*H*-benzo[*d*]imidazol-5-yl)imidazo[1,2-*a*]pyrazine (**18**).

**Figure S36.** ^13^C NMR spectrum of 6-(4-chlorophenyl)-8-(1-cyclohexyl-1*H*-benzo[*d*]imidazol-5-yl)imidazo[1,2-*a*]pyrazine (**18**).

**
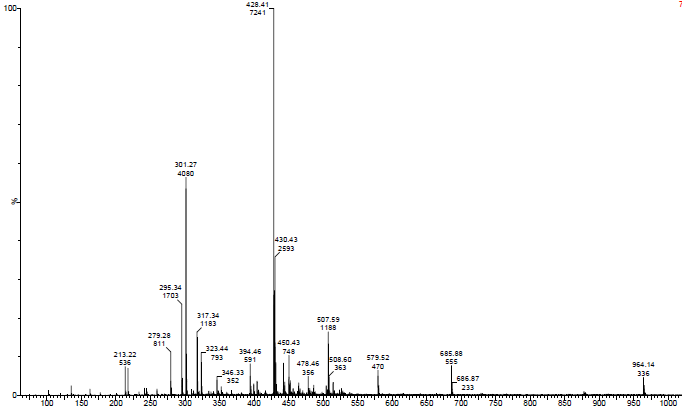
Figure S37.** Mass spectrum of 6-(4-chlorophenyl)-8-(1-cyclohexyl-1*H*-benzo[*d*]imidazol-5-yl)imidazo[1,2-*a*]pyrazine (**18**).

**Figure S38.** ^1^H NMR spectrum of 6-(4-bromophenyl)-8-(1-cyclohexyl-1*H*-benzo[*d*]imidazol-5-yl)imidazo[1,2-*a*]pyrazine (**19**).

**Figure S39.** ^13^C NMR spectrum of 6-(4-bromophenyl)-8-(1-cyclohexyl-1*H*-benzo[*d*]imidazol-5-yl)imidazo[1,2-*a*]pyrazine (**19**).

**
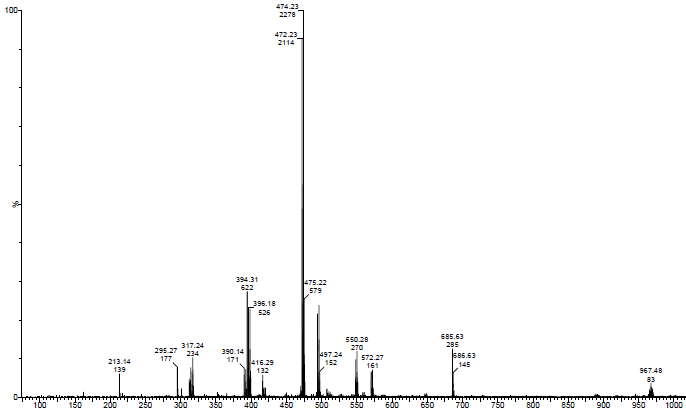
**

**Figure S40.** Mass spectrum of 6-(4-bromophenyl)-8-(1-cyclohexyl-1*H*-benzo[*d*]imidazol-5-yl)imidazo[1,2-*a*]pyrazine (**19**).

**Figure S41.** ^1^H NMR spectrum of 8-(1-cyclohexyl-1*H*-benzo[*d*]imidazol-5-yl)-6-(2-methoxyphenyl)imidazo[1,2-*a*]pyrazine (**20**).

**Figure S42.** ^13^C NMR spectrum of 8-(1-cyclohexyl-1*H*-benzo[*d*]imidazol-5-yl)-6-(2-methoxyphenyl)imidazo[1,2-*a*]pyrazine (**20**).

**Figure S43.** ^1^H NMR spectrum of 8-(1-cyclohexyl-1*H*-benzo[*d*]imidazol-5-yl)-6-(2-fluorophenyl)imidazo[1,2-*a*]pyrazine (**21**).

**Figure S44.** ^13^C NMR spectrum of 8-(1-cyclohexyl-1*H*-benzo[*d*]imidazol-5-yl)-6-(2-fluorophenyl)imidazo[1,2-*a*]pyrazine (**21**).

**Figure S45.** ^1^H NMR spectrum of 8-(1-cyclohexyl-1*H*-benzo[*d*]imidazol-5-yl)-6-(thiophen-2-yl)imidazo[1,2-*a*]pyrazine (**22**).

**Figure S46.** ^13^C NMR spectrum of 8-(1-cyclohexyl-1*H*-benzo[*d*]imidazol-5-yl)-6-(thiophen-2-yl)imidazo[1,2-*a*]pyrazine (**22**).

**Figure S47.** ^1^H NMR spectrum of 8-(1-cyclohexyl-1*H*-benzo[*d*]imidazol-5-yl)-6-(3-(trifluoromethyl)phenyl)imidazo[1,2-*a*]pyrazine (**23**).

**Figure S48.** ^13^C NMR spectrum of 8-(1-cyclohexyl-1*H*-benzo[*d*]imidazol-5-yl)-6-(3-(trifluoromethyl)phenyl)imidazo[1,2-*a*]pyrazine (**23**)

**
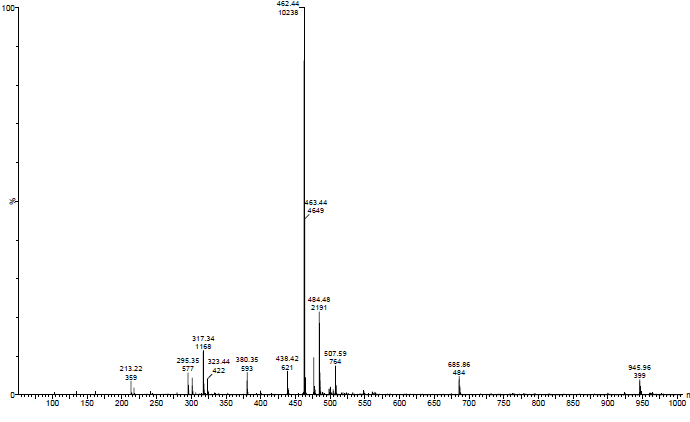
**

**Figure S49.** Mass spectrum of 8-(1-cyclohexyl-1*H*-benzo[*d*]imidazol-5-yl)-6-(3-(trifluoromethyl)phenyl)imidazo[1,2-*a*]pyrazine (**23**)

**Figure S50.** ^1^H NMR spectrum of 6,8-bis(1-cyclohexyl-1*H*-benzo[*d*]imidazol-5-yl)imidazo[1,2-*a*]pyrazine (**30**).

**Figure S51.** ^13^C NMR spectrum of 6,8-bis(1-cyclohexyl-1*H*-benzo[*d*]imidazol-5-yl)imidazo[1,2-*a*]pyrazine (**30**).

**
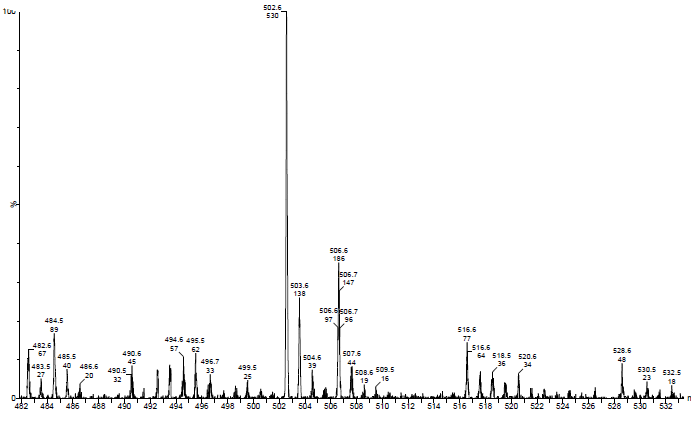
Figure S52.** Mass spectrum of 6,8-bis(1-cyclohexyl-1*H*-benzo[*d*]imidazol-5-yl)imidazo[1,2-*a*]pyrazine (**30**).

**Figure S53.** ^1^H NMR spectrum of 8-(1-cyclohexyl-1*H*-benzo[*d*]imidazol-5-yl)-6-(1-cyclohexyl-1*H*-benzo[*d*]imidazol-6-yl)imidazo[1,2-*a*]pyrazine (**31**).

**Figure S54.** ^13^C NMR spectrum of 8-(1-cyclohexyl-1*H*-benzo[*d*]imidazol-5-yl)-6-(1-cyclohexyl-1H-benzo[d]imidazol-6-yl)imidazo[1,2-*a*]pyrazine (**31**).

**Table S1.** Percent growth inhibition of compounds **8-10**, **12-14**, **22** and **30-31** at single dose concentration of 10 *µ*M

| \| **Cell Panel** \| \| --- \| | **Cell Line** | **8** | **9** | **10** | **12** | **13** | **14** | **22** | **30** | **31** |
| --- | --- | --- | --- | --- | --- | --- | --- | --- | --- | --- | --- |
| Leukemia | CCRF-CEM | -- | 11.65 | 1.19 | 27.20 | -- | 1.10 | -- | 35.14 | 95.72 |
|  | HL-60(TB) | 10.51 | 19.35 | 23.32 | 49.91 | 5.28 | 2.65 | 0.87 | 32.94 | -42.22 |
|  | K-562 | 17.36 | 31.12 | 29.17 | 37.95 | 13.25 | 9.60 | -- | 47.19 | -4.74 |
|  | MOLT-4 | 17.45 | 39.68 | 34.21 | 51.01 | 11.45 | 14.33 | 12.36 | 43.65 | -30.50 |
|  | RPMI-8226 | -- | 18.32 | 14.67 | 38.23 | 6.16 | 20.80 | -- | 52.18 | -37.92 |
|  | SR | 20.65 | 44.60 | 29.66 | 10.86 | 20.78 | 23.63 | 14.96 | 17.05 | 97.53 |
| Non-Small  Cell Lung  Cancer | A549/ATCC | 4.80 | 9.00 | 7.33 | 36.17 | 8.95 | -- | 1.63 | 4.93 | -44.85 |
|  | EXVX | 10.34 | 2.51 | 7.85 | 29.58 | -- | -- | 3.54 | 9.56 | -13.11 |
|  | HOP-62 | 12.98 | 12.94 | 3.33 | 13.19 | 13.25 | 7.41 | 0.44 | 20.82 | -55.40 |
|  | HOP-92 | 21.90 | 10.87 | 17.39 | NT | 7.05 | 24.98 | -- | NT | NT |
|  | NCI-H226 | 3.73 | -- | -- | 17.85 | 7.09 | -- | -- | 13.10 | -56.37 |
|  | NCI-H23 | 8.76 | -- | -- | 9.02 | 4.49 | 8.60 | 5.56 | 4.61 | -41.51 |
|  | NCI-H322M | 10.94 | 2.91 | -- | 8.38 | -- | 1.19 | 0.45 | -- | 98.77 |
|  | NCI-H460 | 3.26 | -- | 2.90 | 7.35 | -- | 7.12 | -- | 1.35 | -48.51 |
|  | NCI-522 | 15.24 | 16.06 | 18.50 | 33.03 | 22.62 | 10.40 | 14.55 | 17.66 | -52.60 |
| Colon  Cancer | COLO 205 | -- | 0.28 | -- | -- | -- | -- | -- | -- | -64.36 |
|  | HCC-2998 | 3.13 | -- | -- | -- | -- | -- | -- | -- | -93.43 |
|  | HCT-116 | 6.06 | 3.81 | 18.60 | 23.68 | 6.58 | 15.85 | -- | 15.88 | -61.35 |
|  | HCT-15 | 4.25 | 19.09 | 11.86 | 15.26 | 4.85 | 18.81 | 6.48 | 26.01 | -11.46 |
|  | HT29 | 4.48 | 9.21 | -- | 16.53 | 8.33 | 9.20 | -- | 28.13 | -26.16 |
|  | KM12 | -- | 5.70 | 2.27 | 8.69 | 7.30 | 11.72 | 6.84 | 56.20 | -61.09 |
|  | SW-620 | -- | -- | -- | 1.43 | -- | -- | -- | -- | -3.82 |
| CNS  Cancer | SF-268 | 5.80 | 1.33 | 3.96 | 11.82 | 5.13 | 5.01 | 2.5 | 4.85 | -67.02 |
|  | SF-295 | 7.58 | -- | 12.47 | 7.88 | -- | -- | -- | 10.42 | -86.52 |
|  | SF-539 | 6.65 | 2.17 | 9.71 | 7.72 | -- | 2.82 | -- | 25.38 | -82.05 |
|  | SNB-19 | 10.53 | 1.72 | 4.26 | 16.69 | -- | 3.96 | -- | 5.02 | 93.37 |
|  | SNB-75 | 16.41 | 5.65 | 15.94 | 9.15 | -- | -- | -- | 6.25 | -89.61 |
|  | U251 | -- | 4.44 | 3.40 | 27.93 | 3.57 | 30.19 | -- | 29.66 | -83.28 |
| Melanoma | LOX IMVI | 9.14 | 4.86 | 7.86 | 11.39 | 0.76 | 14.54 | 1.01 | 11.71 | -87.39 |
|  | MALME-3M | -- | -- | -- | -- | -- | 3.01 | -- | 8.16 | -14.31 |
|  | M14 | -- | 1.61 | 9.96 | 0.39 | 0.73 | 3.43 | -- | -- | -50.79 |
|  | MDA-MB-435 | -- | 0.53 | 6.92 | 4.07 | -- | 6.00 | -- | -- | -72.96 |
|  | SK-MEL-2 | 0.53 | -- | 9.88 | 20.60 | 6.30 | 0.58 | 13.14 | NT | -65.61 |
|  | SK-MEL-28 | -- | -- | 4.86 | -- | -- | -- | -- | -- | -19.48 |
|  | SK-MEL-5 | 8.35 | 5.71 | 5.95 | 2.13 | 2.90 | 24.80 | 1.16 | 20.50 | -98.42 |
|  | UACC-257 | -- | -- | -- | 35.53 | -- | -- | -- | 5.79 | -71.51 |
|  | UACC-62 | 16..41 | 2.01 | 1.25 | 20.96 | 2.44 | 18.51 | 0.23 | 12.43 | -47.66 |
| Ovarian  Cancer | IGROV1 | 8.45 | -- | 5.82 | 3.68 | 6.40 | 19.06 | -- | -- | -41.94 |
|  | OVCAR-3 | -- | -- | -- | 4.29 | -- | -- | 0.91 | -- | -85.15 |
|  | OVCAR-4 | 9.25 | -- | 7.51 | 15.02 | -- | -- | -- | -- | 97.16 |
|  | OVCAR-5 | -- | -- | 1.45 | -- | -- | -- | -- | -- | -24.00 |
|  | OVCAR-8 | 3.55 | -- | 7.75 | 23.99 | 3.09 | 6.38 | -- | 11.57 | -24.11 |
|  | NCI/ADR-RES | 5.47 | -- | 1.93 | 11.83 | 2.36 | 4.17 | 6.79 | 4.12 | 98.89 |
|  | SK-OV-3 | 6.59 | -- | 5.37 | -- | -- | -- | -- | 11.77 | -71.86 |
| Renal  Cancer | 786-0 | 1.30 | 8.24 | 12.65 | 13.33 | 3.12 | -- | 0.59 | 13.93 | -47.08 |
|  | A498 | 3.51 | -- | -- | -- | NT | -- | NT | -- | -88.07 |
|  | ACHN | -- | -- | 10.91 | 8.22 | -- | 4.32 | -- | 4.08 | -58.83 |
|  | CAKI-1 | NT | NT | NT | 20.67 | -- | -- | -- | 9.21 | 82.80 |
|  | RXF 393 | -- | 23.31 | -- | 0.15 | 35.96 | -- | -- | 4.30 | -89.06 |
|  | SN12C | -- | 4.83 | -- | 13.57 | -- | 6.33 | -- | 13.88 | -54.59 |
|  | TK-10 | -- | -- | -- | 5.82 | -- | -- | -- | -- | -59.30 |
|  | UO-31 | 40.12 | 20.86 | 33.47 | 37.85 | 22.83 | 23.08 | 16.37 | 26.76 | -85.42 |
| Prostate  Cancer | PC-3 | 10.26 | 13.53 | 22.63 | 13.28 | 10.23 | 17.92 | 7.16 | 27.87 | -64.10 |
|  | DU-145 | -- | 2.64 | -- | 6.09 | -- | -- | 3.63 | 6.22 | -59.08 |
| Breast  Cancer | MCF7 | 37.62 | 15.85 | 19.68 | 35.71 | 9.59 | 11.97 | 34.03 | 23.85 | -3.78 |
|  | MDA-MB-231/ATCC | 3.12 | 6.64 | -- | 18.16 | -- | 7.65 | 7.47 | 16.80 | -68.66 |
|  | HS 578T | 2.68 | -- | 5.69 | 4.25 | -- | 6.34 | -- | 18.91 | 96.60 |
|  | BT-549 | 6.38 | 0.43 | 8.55 | 1.57 | -- | 4.68 | -- | 5.90 | -48.93 |
|  | T-47D | 25.30 | 25.52 | 46.21 | 22.02 | 10.08 | 54.44 | 37.28 | 34.66 | -17.65 |
|  | MDA-MB-468 | 6.27 | -- | -- | 5.23 | -- | 0.73 | 28.31 | 3.28 | -20.24 |

-- indicates GI < 1%; NT, not tested; - (negative) lethal to cancer cells


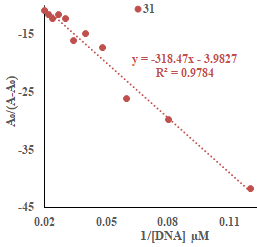


**Figure S55.** Benesi-Hildebrand plot of compound **31** {A_o_/(A-A_o_) vs. 1/[DNA]} for absorption spectrum on incremental addition of CT-DNA.

| 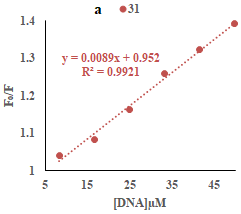 | 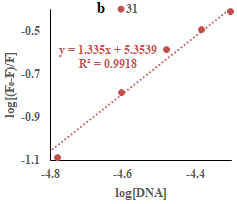 |
| --- | --- |

**Figure S56.** (a) Stern–Volmer plot (F₀/F versus [DNA]) and (b) Scatchard plot {log(F₀-F)/F versus log [DNA]} for interaction of compound **31** with CT-DNA.

**
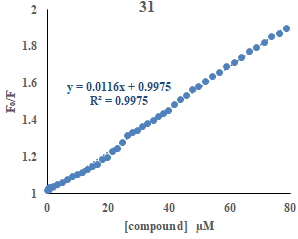
**

**Figure S57.** Stern–Volmer plot (F₀/F versus [compound **31]**) for interaction of EtBr-CT-DNA complex with compound **31**

| 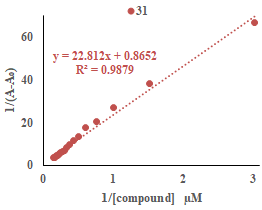 |
| --- |

**Figure S58.** Benesi-Hildebrand plot {A_o_/(A-A_o_) vs. 1/[DNA]} for interaction of BSA with compound **31**

| 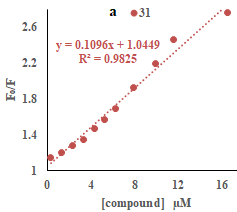 | 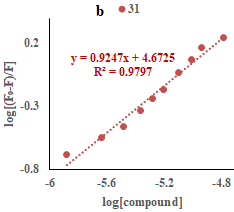 |
| --- | --- |

**Figure S59.** (a) Stern–Volmer plots (F₀/F versus [compound]) and (b) Scatchard plots {log(F₀-F )/F versus log [compound]} for interaction of BSA with compound **31**

| 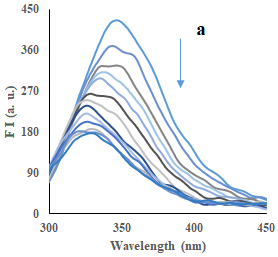 | 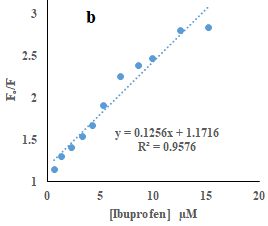 |
| --- | --- |

**Figure S60.** (a) Electronic spectrum of compound **31**-BSA complex with incremental addition of ibuprofen and (b) Stern–Volmer plot (F₀/F versus [ibuprofen]) for interaction of ibuprofen with compound **31**-BSA complex

**Table S2.** The docking results based on the binding free energies (kcal/mol) of compound **31** docked into 1BNA and RMSD from the co-crystallized ligand

| **Mode** | **Affinity (kcal/mol)** | **RMSD (Å)** |
| --- | --- | --- |
| 1 | -11.1 | 0.000 |
| 2 | -10.6 | 2.139 |
| 3 | -10.3 | 21.607 |
| 4 | -9.9 | 3.007 |
| 5 | -9.7 | 3.486 |
| 6 | -9.4 | 24.108 |
| 7 | -9.4 | 2.817 |
| 8 | -9.3 | 25.491 |
| 9 | -9.3 | 2.280 |

**References**

1. Maya, F. & Tour, J.M. Synthesis of terphenyl oligomers as molecular electronic device candidates. *Tetrahedron*, **60,** 81-92 (2004).
2. Singh, I., Luxami, V. & Paul, K. Effective synthesis of benzimidazoles-imidazo[1, 2-*a*]pyrazine conjugates: A comparative study of mono-and bis-benzimidazoles for antitumor activity.  *Eur. J. Med. Chem.* **180**, 546-561 (2019).
